# Catechol-Amine-Decorated Epoxy Resin as an Underwater Adhesive: A Coacervate Concept Using a Liquid Marble Strategy

**DOI:** 10.1021/acsomega.2c04163

**Published:** 2023-02-16

**Authors:** Monisha Baby, Soumyamol Panthaplackal Bhaskaran, Satheesh Chandran Maniyeri

**Affiliations:** † Cochin University of Science and Technology, Ernakulam 682022, Kerala, India; ^‡^Analytical and Spectroscopic Division and ^§^Polymers and Special Chemical Division, Vikram Sarabhai Space Centre, Thiruvananthapuram 695022, Kerala, India

## Abstract

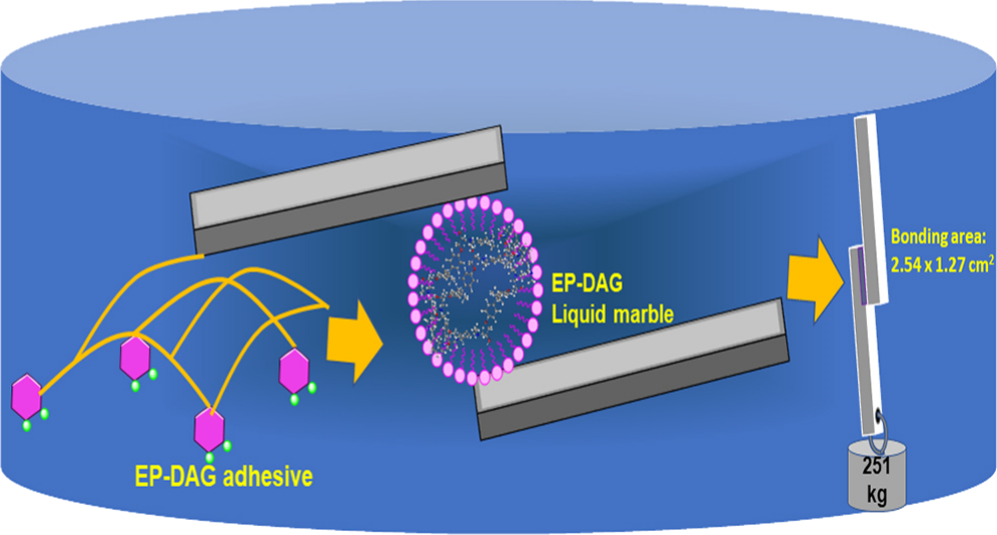

The attachment phenomena of various hierarchical architectures found in nature, especially underwater adhesion, have drawn extensive attention to the development of similar biomimicking adhesives. Marine organisms show spectacular adhesion characteristics because of their foot protein chemistry and the formation of an immiscible phase (coacervate) in water. Herein, we report a synthetic coacervate derived using a liquid marble route composed of catechol amine-modified diglycidyl ether of bisphenol A (EP) polymers wrapped by silica/PTFE powders. The adhesion promotion efficiency of catechol moieties is established by functionalizing EP with monofunctional amines (MFA) of 2-phenyl ethylamine and 3,4-dihydroxy phenylethylamine (DA). The curing activation of MFA-incorporated resin pointed toward a lower activation energy (50.1–52.1 kJ mol^–1^) compared with the neat system (56.7–58 kJ mol^–1^). The viscosity build-up and gelation are faster for the catechol-incorporated system, making it ideal for underwater bonding performance. The PTFE-based adhesive marble of the catechol-incorporated resin was stable and exhibited an adhesive strength of 7.5 MPa under underwater bonding conditions.

## Introduction

Underwater adhesives are inevitable for submerged substrates and biomedical implants. At the same time, they present several technical challenges.^1−3^ Despite the wide acceptance of synthetic adhesives under dry conditions, they fail to perform in water often due to moisture infiltration.^4−8^ The presence of moisture causes poor adhesive performance in a dual manner, viz, by plasticization of reactive components, thereby reducing the extent of curing, leading to a reduction in adhesive strength, and as a barrier layer curtailing the proximate interaction at the adhesive/substrate interface.^9−11^ Generally, silicone adhesives are the primary choice for water-resistant applications because of their hydrophobic characteristics.^12^ However, this class of adhesives is also not devoid of limitations, for example, lower adhesive strength. Hence, the modification of adhesive strength is indispensable to meet the growing demand for underwater adhesives.

A perfect remedy for underwater bonding is revealed by marine organisms such as mussels and sandcastle worms, which exhibit fascinating adhesion chemistry derived from specialty foot proteins and the consecutive setting mechanism under marine pH conditions.^13−15^ The major molecule responsible for the firm adhesion is 3,4-dihydroxy phenylalanine (l-Dopa).^16^ These natural underwater welders explicitly provide clues to boost research in this area. The critical mechanistic/crosslinking pathways include the formation of a water-immiscible adhesive liquid (coacervates), wetting on the substrate by replacing the water layer, fast-setting mechanisms, and the insolubility of the cured network in water.^1,17,18^ Oppositely charged polyelectrolytes in the foot protein act as coacervates, and their formation depends on different factors such as pH, ionic strength, and polyelectrolyte concentrations.^19−23^l-Dopa is proved to be a connective/interacting species on the substrates, and l-Dopa-incorporated synthetic polymers displayed strong interface interaction.^24−26^ The alkaline pH and metal ions in the sea environment act as triggers for crosslinking and solidification of the adhesive.^13,27^

In the domain of synthetic mimics of coacervates, a tannic acid-incorporated acrylate adhesive is reported for improved underwater adhesion because of the presence of numerous pyrogallol and catechol groups.^28^ In another report, a tannic acid-based coacervate is reported for underwater adhesives for biomedical applications.^20^ The formation of underwater-performing complex coacervates was reported to be attained by mixing two oppositely charged graft copolymers functionalized with thermoresponsive poly(N-isopropylacrylamide) chains that showed good underwater adhesion compared with standard commercial pressure-sensitive adhesives (*W*_adh_ = 0.02–0.26 J m^–2^) and adhesion performance similar to other biomimetic underwater adhesives (*W*_adh_ = 0.75–6.5 J m^–2^).^24^ Multiphase coacervates were formulated by introducing poly(ethylene glycol) diacrylate into a synthetic polyelectrolyte and crosslinking to form an adhesive network with an underwater adhesion strength of 1.2 MPa, which is four times higher than that of the sandcastle worm.^29^ Coacervate-based underwater adhesives were also reported under physiological conditions for biomedical applications.^30,31^ Xu et al. reported a pioneering work of epoxy-catechol amine-based antifouling coating.^32^ Recently, Li et al. reported epoxy-dopamine-based primers for SS substrates, with improved performance under wet conditions as well as after exposure to hot water.^33^ In our earlier study, we reported dopamine-functionalized epoxy primers as moisture-resistant coatings with an underwater bonding strength of 7–7.5 MPa.^34^ North et al. reported poly(catechol-styrene)-based adhesives with the highest reported underwater bonding strength of 3 MPa on an Al substrate.^35^

In this study, we demonstrated a novel liquid marble technology as an efficient way to mimic the formation of coacervates that in turn help achieve strong underwater adhesion. Liquid marbles (LMs) are artificially synthesized by stabilizing liquid resins with solid particles.^36^ LMs are explored in different areas of material science, including pressure-sensitive adhesives, miniature reactors, microfluidics, delivery carrier materials, etc. This study attempts to extend the concept of LMs to a novel application area of underwater adhesion, which could have great application potential. The formation and stability of LMs depend on various factors such as their formation energy and parameters such as the surface tension of the liquid as well as the type of solid particles, the size of LMs, the viscosity of the liquid, the particle size, etc.^37^ Toward this, monofunctional amine-decorated epoxy polymers were realized by partially reacting diglycidyl ether of bisphenol A (EP) with dopamine (DA) or 2-phenyl ethyl amine (PEA) and crosslinking with a difunctional aliphatic amine (G). The role of catechol in imparting better underwater adhesiveness was confirmed by comparing with non-catechol-based 2-phenyl ethyl amine (PEA). The synthesized polymers were studied for their underwater adhesion, viz., as such, by blending with fillers, and through the liquid marble technology using silica nanoparticles and PTFE powder. The innovative method through the adhesive marble technology demonstrated herein can be a good overreaching strategy for adhesion under submarine/wet conditions.

## Experimental Section

### Materials

Dopamine hydrochloride (DAHCl, purity 99%) and 2-phenylethyl amine were supplied by Sigma-Aldrich. Diglycidyl ether of bisphenol A (EP, epoxy equivalent weight 178.5 g) was purchased from Huntsman Pvt. Ltd. Methanol, acetone, trichloroethylene (TCE), and methyl ethyl ketone (MEK) of 99.5% purity were supplied by Merck Specialties Pvt Ltd, Germany. Gaskamine 328 was purchased from Mitsubishi Gas Chemical, Japan. Silica nanopowder (20 nm) and PTFE (1 μm) powder were purchased from Sigma-Aldrich.

### Instrumental Techniques

The molar mass distribution and average molar mass of the samples were determined using a Waters 600 GPC equipped with a Waters 2414 refractive index detector. FTIR spectra were recorded with a PerkinElmer spectrum GXA spectrophotometer in the wavenumber range of 400 to 4000 cm^–1^. ^1^H and ^13^C NMR spectra were recorded using a Bruker Advance spectrometer (300 MHz). Thermogravimetric analysis (TGA) was performed using a TA instruments, model SDT-2960 simultaneous DTA (differential thermal analysis)–TGA at a heating rate of 10 °C/min under a N_2_ atmosphere. The lap shear strength (LSS) of specimens was evaluated as per ASTM D 1002 using an Instron UTM 5569 Microtest model EM2/50/FR at a crosshead speed of 10 mm/min. Tensile properties of specimens were measured as per ASTM D638-14 using a Universal Testing Machine at a crosshead speed of 10 mm/min. Dumbbell specimens were prepared according to ASTM standard Type V. The fracture toughness was determined as per ASTM D5045-14 using single edge notch bend (SENB) specimens in a three-point bending mode at a crosshead speed of 10 mm/min. Dynamic mechanical properties of the composite specimens were determined using a TA instruments DMA Q800 (TA Instruments, DE) in the three-point bending mode. Rheological analysis was performed by TA Instruments, Hybrid rheometer model Discovery HR-3, at an isothermal temperature of 30 °C at 1% strain and a frequency of 1 Hz using aluminum parallel-plate geometry (25 mm diameter).

### Synthesis of EP-DA and EP-PEA

DAHCl (0.5 g, 2.7 mmol) was deprotected by stirring with basic alumina (0.8 g) in methanol (25 mL) for 10 min and filtered. This filtrate was taken in a round-bottom flask equipped with a N_2_ inlet and magnetic stirring and was reacted with EP (10 g, 28 mmol) and 25 mL of acetone at 40 °C for 12 h to obtain the linear polymer EP-DA. For the linear polymer EP-PEA, 0.5 g (4.12 mmol) of PEA was reacted with EP (10 g, 28 mmol) in 25 mL of acetone. After the reaction, excess solvent was distilled off under reduced pressure to obtain pale yellowish resin products (EP-DA and EP-PEA).

### Crosslinking Reaction

EP-DA and EP-PEA reacted with Gaskamine 328 (GK) in the stoichiometric ratio of epoxy and amine equivalents. Ten grams of EP-PEA, EP-DA, and EP reacted with 2.6, 2.5, and 3.08 g of G, respectively, to form EP-PEAG, EP-DAG, and EP-G.

### Sample Preparation before Bonding

The area for bonding (1.3 × 2.5 cm^2^) of Al (alloy AA 2024 coupons) substrates of dimension 10 × 2.5 × 0.3 cm^3^ was cleaned with TCE and MEK, roughened using an emery paper of grit size 80 in 45° cross abrading, followed by chromic acid etching by dipping the specimens at 70 °C for 15 min. The chromic acid solution was prepared by the Forest Physical Laboratory procedure.^38^ Steel and copper specimens for dry bonding and Al for underwater bonding applications were prepared by roughening with emery paper of grit size 80 in 45° cross abrading, followed by cleaning by MEK, and drying at 70 °C for 15 min.

### Determination of the Swelling Index and Gel Content

A sample (20 mg, *m*_0_) of PGDX was immersed in 20 mL of solvents (methanol, toluene, and water used in this study) separately for 24 h. After 24 h, the solvent was decanted, samples were wiped with tissue paper, and the weight was determined (*m*_1_). The swelling index was calculated using eq 1. The samples after 24 h immersion in solvents were dried in an oven at 100 °C for 8 h to determine the final weight (*m*_2_). The gel content was calculated using eq 2 ^39^
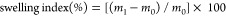
1
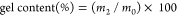
2

## Results and Discussion

**Scheme 1:**
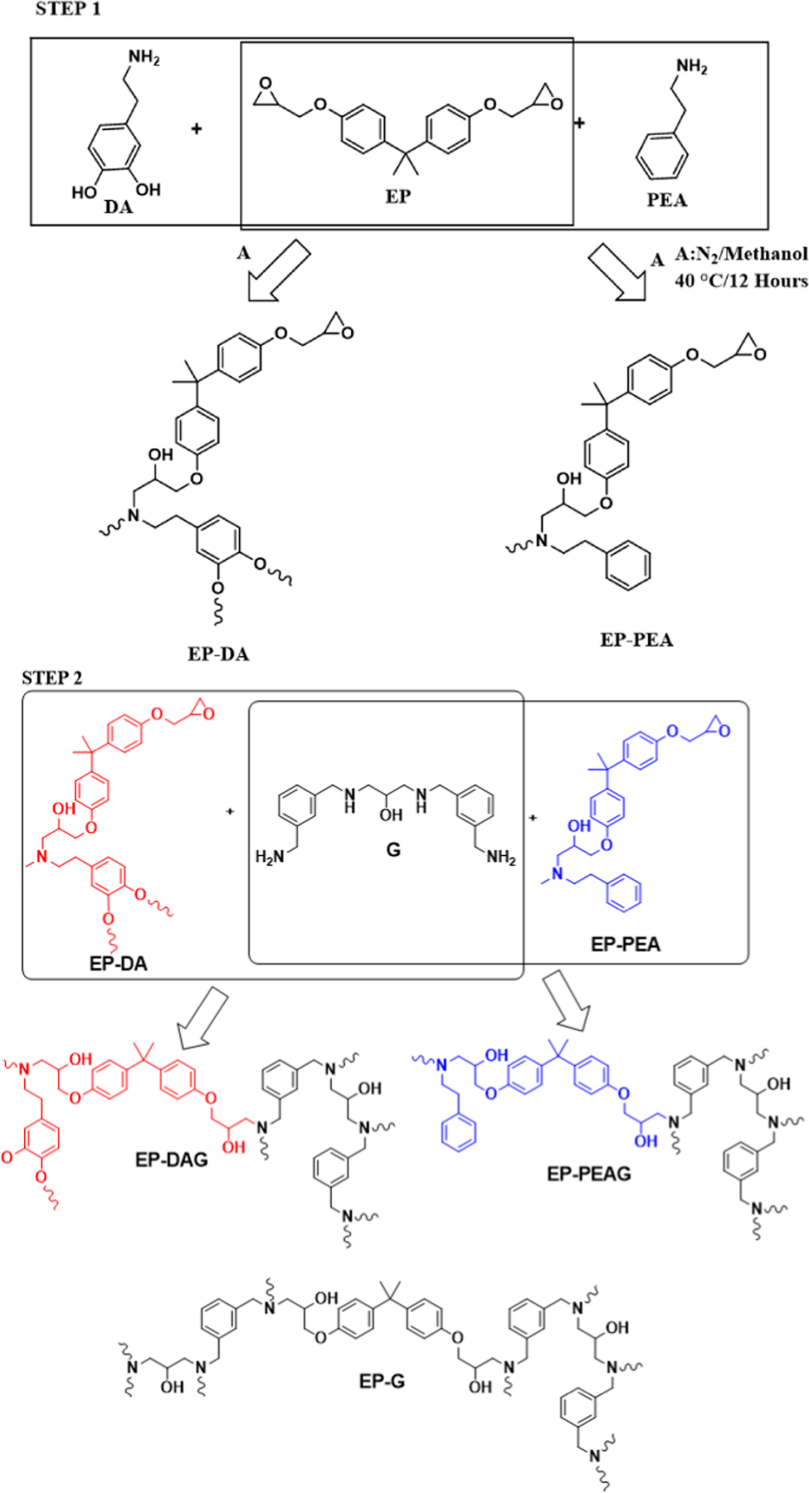
Synthesis of Linear Polymers EP-DA, EP-PEA, and EP-DAG and Crosslinked Polymers EP-DAG, EP-PEAG, and EP-G

Linear polymers with DA and PEA were synthesized by partially reacting EP with DA and PEA (Scheme 1, step 1). The properties of the synthesized polymers (EP-DA and EP-PEA) were compared with those of the neat epoxy resin (EP) by curing with Gaskamine 328 (G), as shown in Scheme 1, step 2.

**Figure 1:**
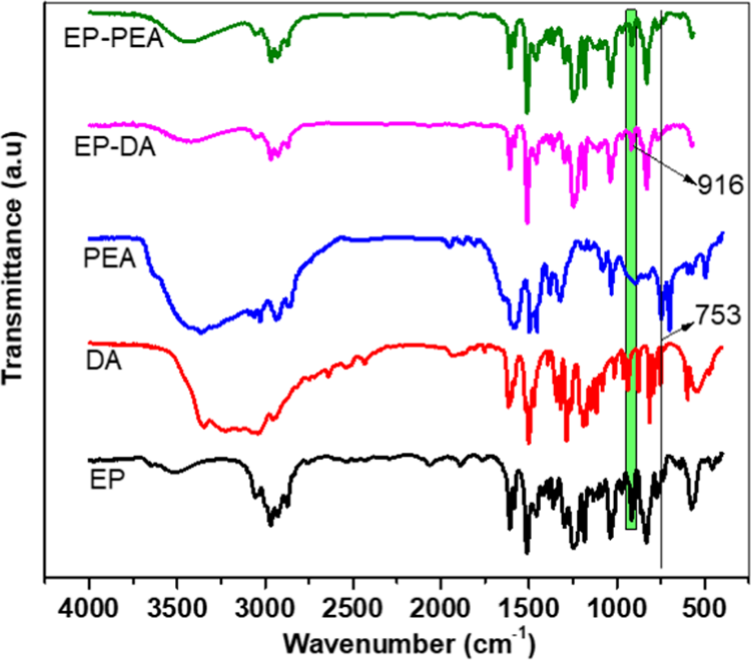
FTIR spectra of EP, DA, PEA, EP-DA, and EP-PEA.

The complete reaction of DA and PEA with EP was confirmed from the FTIR spectra (Figure 1) as the peak corresponding to amine at 753 cm^–1^ for EP-DA and EP-PEA was absent. The epoxy peak intensity at 915 cm^–1^ is compared with aromatic C=C vibrations at 1509 cm^–1^, which is considered as the constant peak for EP, EP-DA, and EP-PEA. Upon comparison with the aforementioned peak, the epoxy peak intensity for EP is reduced to 52–58% for EP-DA and PEA, clearly revealing the partial reaction of epoxy groups of EP with DA and PEA (Figure S1). A decrease in the epoxy values for EP-PEA (4.8 equiv/kg) and EP-DA (4.1 equiv/kg) systems compared with the neat system (EP, 5.6 equiv/kg) confirmed the partial reaction of DA and EP. It can be inferred that the effective role played by the catechol hydroxyl groups could have been responsible for the decrease in the epoxy value noted for EP-DA compared with EP-PEA, leading to the increased extent of reaction by homopolymerization (Scheme S1). The epoxy value of 4.8 equiv/kg for EP-PEA essentially meant that for one molecule of PEA, two epoxy groups are incorporated as per the reaction conditions described in the Experimental Section. A significant reduction in the epoxy value is noted after 90 days, clearly proving the accelerating effect of tertiary amines on the homopolymerization of epoxy. In the case of EP-DA, the epoxy value of 4.1 equiv/kg indicates that 4.5 units of epoxy groups are incorporated per one molecule of DA by the cumulative acceleration effect of -OH and tertiary amine groups. The schematic representation of acceleration by monofunctional amines (MFAs) is given in Scheme S1. The epoxy value of EP-PEA is decreased to 2.6 equiv/kg after a duration of 90 days, whereas EP-DA turned into an insoluble solid mass (Figure S2). Compared with EP-PEA, the lower epoxy value for EP-DA emphasized a higher reactivity of DA. The molar mass (Mn) values of EP-DA, EP-PEA, and EP were 1774, 896, and 246 g/mol, respectively, indicating the additional molecular weight build-up upon reacting with DA and PEA (Table S1). Further evidence of the reaction process is confirmed from the variation in the elemental percentage by the reaction of DA and PEA leading to an increase in the percentage of nitrogen in EP-DA and EP-PEA compared with EP (Table S2).

The ^1^H NMR spectra (Figure S3) show that the aromatic proton peak at 6.4–6.6 ppm for DA and at 7.2–7.4 ppm for PEA shifted to 6.9–7.1 ppm, confirming the reaction of the amine hydrogen with epoxy. The peaks corresponding to glycidyl protons were noted at 2.2–4.16 ppm for EP and MFA-incorporated EP.

### Cure Kinetics

**Figure 2:**
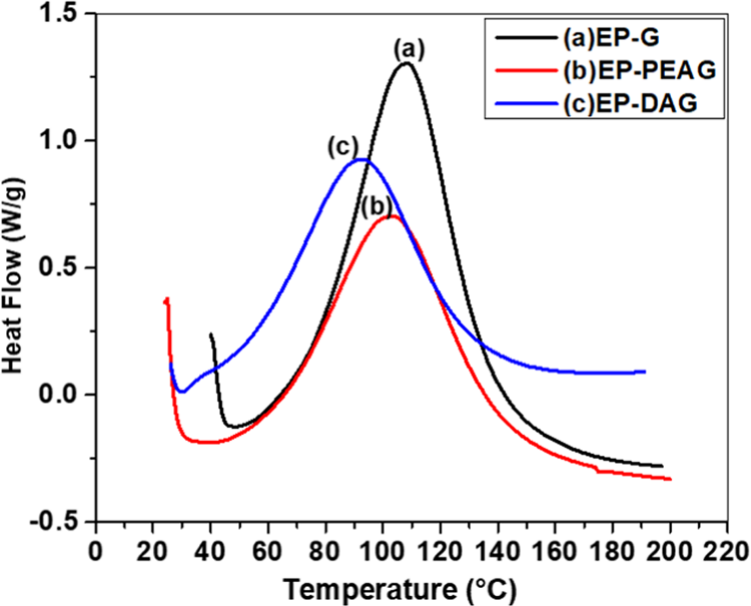
DSC thermograms of (a) EP-DAG, (b) EP-PEAG, and (c) EP-G.

DSC thermograms of EP, EP-PEA, and EP-DA with G showed a shift in the cure profile toward a lower temperature regime for the monofunctional amine-incorporated system (Figure 2).

The cure initiation temperature (*T*_i_), peak temperature (*T*_p_), and final temperature (*T*_f_) (Figure 2, Table 1) of the MFA-incorporated system were found to be lower, clearly indicating the cure acceleration proficiency of EP-PEAG and EP-DAG systems compared with EP-G. Among DA and PEA, the early cure initiation of EP-DAG indicated a better acceleration effect for DA. The total enthalpy of reaction (Δ*H*) followed a decreasing trend in the order EP-G > EP-PEAG > EP-DAG, which is direct evidence of partial reaction of MFA with EP-PEAG and EP-DAG. To further establish the cure pathways, kinetics of the curing was evaluated using DSC at heating rates of 5, 10, 15, and 20 °C (Figure S4). The thermogram peaks showed a shift to a higher temperature regime with increasing heating rate. This is due to the limited time available for the reaction to occur at the required specific temperature corresponding to the particular curing reaction.^40^ The Kissinger and Flynn–Wall–Ozawa kinetic models were followed for the curing kinetics studies.

**Table 1:** Parameters Calculated from Differential Scanning Calorimetry

system	*T*_i_ (°C)	*T*_p_ (°C)	*T*_max_ (°C)	Δ*H* (J/g)
EP-G	49	109	182	391
EP-PEAG	36	104	179	296
EP-DAG	30	93	164	276

**Figure 3:**
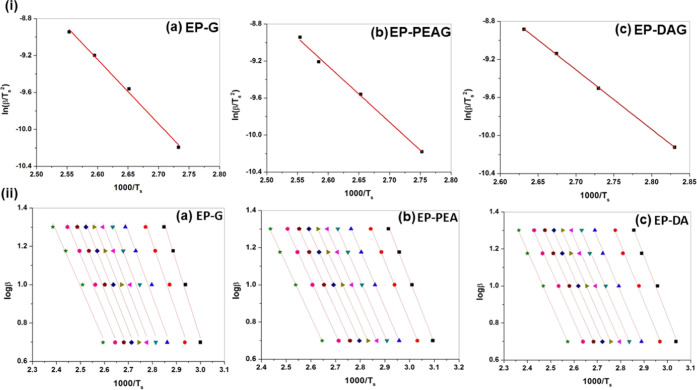
(i). Kissinger plots and (ii) Flynn–Wall–Ozawa plots of EP-G, EP-PEAG, and EP-DAG.

Equation 3 is conventionally used for the activation energy (*E*_a_) determination by the Kissinger method based on nonisothermal kinetics
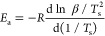
3where *R* is the universal gas constant (8.314 J mol^–1^ K), *T*_s_ is the DSC peak temperature, ‘β’ is the heating rate, and *E*_a_ is the activation energy, represented by the slope of the straight line obtained by plotting ln (β/*T*_s_^2^) against 1000/Ts (Figure 3i).

As the evaluation of a single activation energy for the complete curing reaction does not give the whole idea about the curing behavior of the reaction, the Flynn–Wall–Ozawa (FWO) method, which is based on the approximation that at a given degree of conversion, the reaction rate is only a function of temperature, was also used for kinetic parameter determination. Using this method, for different heating rates at a constant degree of conversion, α(*T*), a linear relationship is observed by plotting log β versus 1/*T*, and the activation energy (*E*_a_) is represented by the slope of the straight line. Conversion ranges from 0.05 to 0.90 were investigated. The FWO plots for the three systems are given in Figure 3ii.

**Figure 4:**
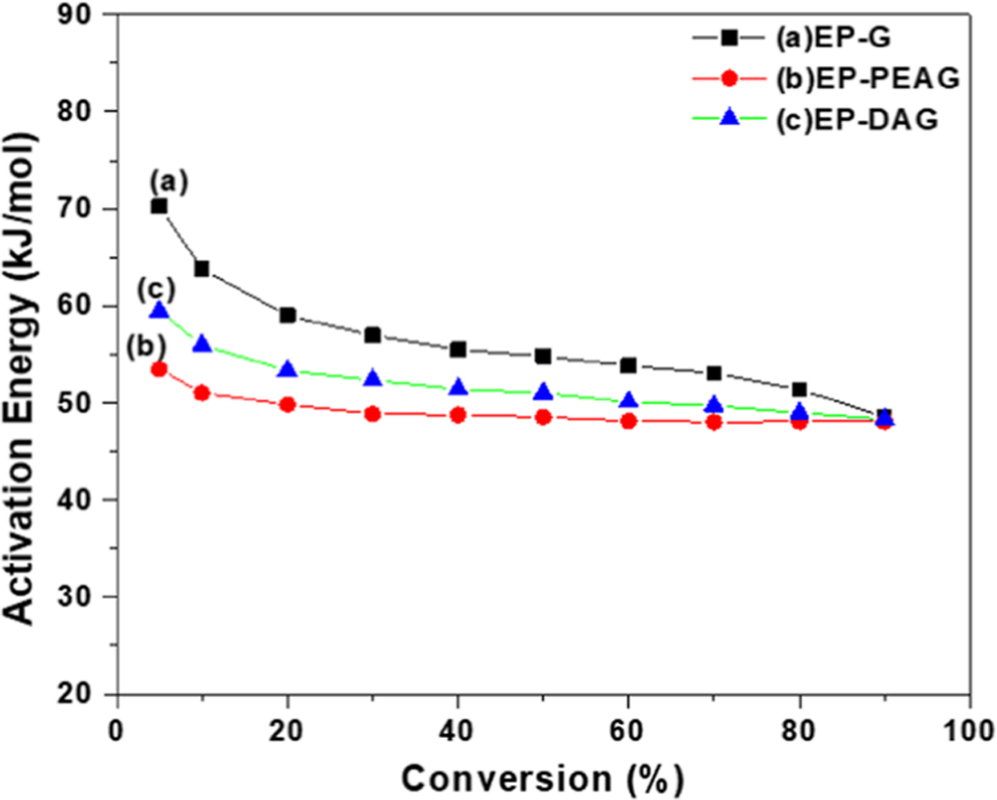
Variation of the activation energy with percentage conversion.

By this method, the activation energy can be obtained using eq 4
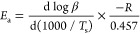
4where *T*_s_ is the peak temperature of the curing exotherm, *R* is the universal gas constant (8.314 J mol^–1^ K), and β is the heating rate. *E*_a_ values calculated using the two methods adopted are presented in Table 2. As expected, the *E*_a_ values were higher for the MFA-incorporated system when compared with the neat adhesive (EP-G), confirming the activation effect of DA and PEA. Interestingly, the percentage of conversion by FWO indicated a decrease in *E*_a_ with respect to an increase in the conversion percentage, revealing the autocatalytic effect of epoxy systems (Figure 4).

**Table 2:** Average Cure Activation Energy for the Three Systems

system	*E*_a_ (kJmol^–1^) (Kissinger Method)	*E*_a_ (kJmol^–1^) (Flynn–Wall–Ozawa method)
EP-G	58	56.7
EP-PEAG	50.5	50.1
EP-DAG	52.1	52.1

The DSC thermogram of the EP-G-cured sample (Figure S5i) exhibited residual cure (∼30 J/g) compared with EP-PEAG and EP-DAG systems. The FTIR spectra of the cured samples also confirmed a comparatively higher residual curing for EP-G compared with EP-PEAG and EP-DAG from the epoxy peak intensity at 915 cm^–1^ (Figure S5ii). The resin system curing was conducted at 60 °C (for the EP-G system), and the associated structural changes were noted at different time intervals (0, 20, and 45 min) by FTIR spectroscopy; the changes in the intensity of the epoxy peak and -NH peaks point toward the epoxy–amine reaction (Figure S6). To evaluate the effect of hydrogen bonding, we conducted computational studies using the PM-6 method for the EP-G system. The hydrogen bonds present in the system include N-HO- at 2.37 Å, -O-HO- at 2.40 Å, and -OH-OH at 1.91 and 2.19 Å, as shown in Figure S7. This observation unequivocally confirms the role of MFA in cure acceleration, leading to fast cure completion. The thermal stability (Figure S8) also confirmed a similar trend for EP-G, EP-PEAG, and EP-DAG systems. The lower thermal stability (*T*_max_) noted for EP-DAG than for EP-PEAG and EP-G could have resulted from the formation of a higher extent of ether-linked polymeric chains by homopolymerization.

### Rheological Characteristics

**Figure 5:**
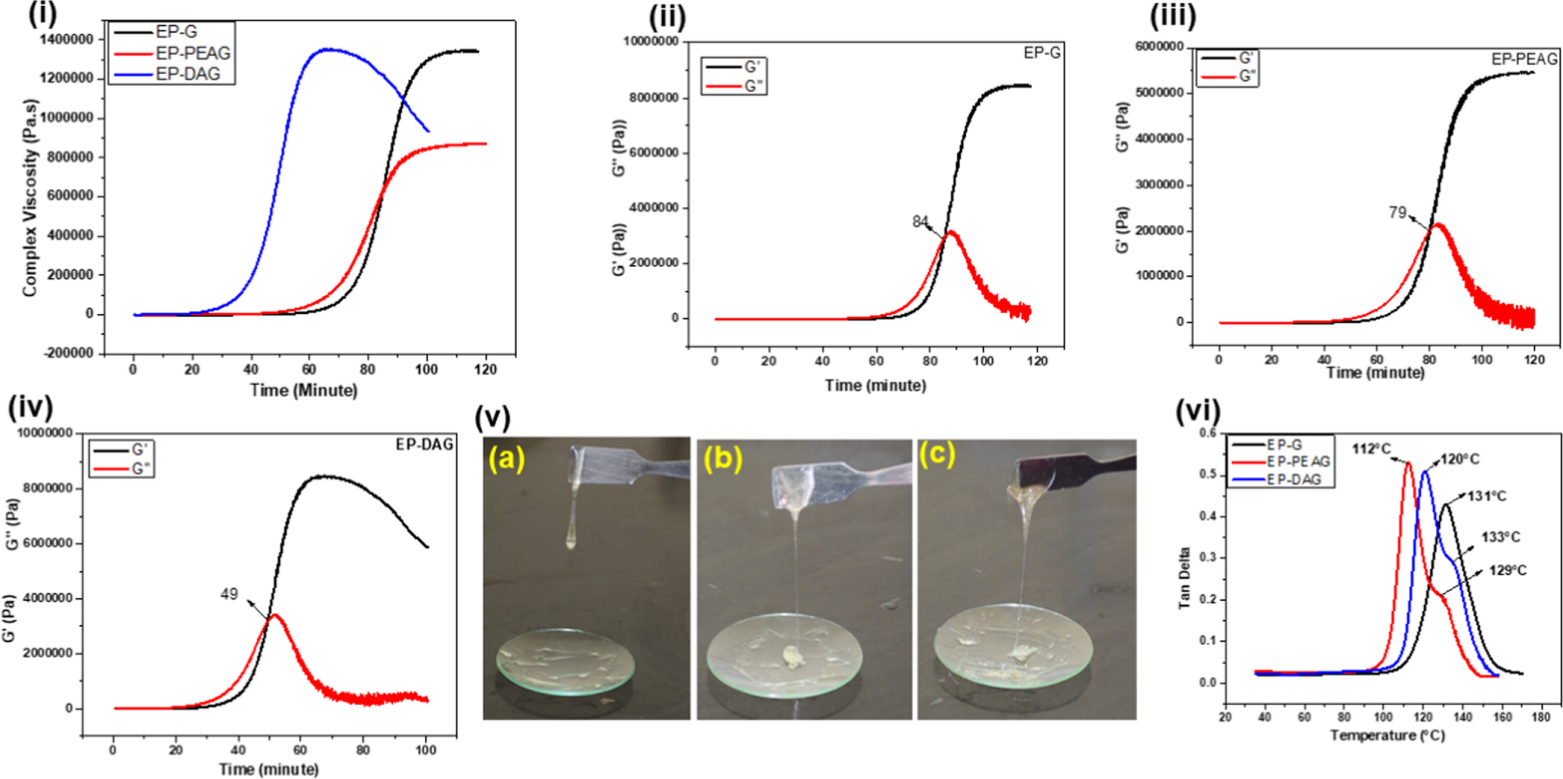
(i). Rheology of EP-G, EP-PEAG, and EP-DAG. Gel points of (ii) EP-G, (iii) EP-PEAG, and (iv) EP-DAG. (v) Digital images of (a) EP-G, (b) EP-PEAG, and (c) EP-DAG. (vi) DMA curves of different systems.

Given the need to prevent the miscibility of the polymeric network with water, the fast curing of EP-DAG compared with the other systems is highly desirable for wet bonding applications. As expected, the isothermal rheogram at 30 °C (Figure 5i) exhibited a higher viscosity build-up for EP-DAG and EP-PEAG systems compared with the neat adhesive (EP-G). The gel point of the adhesive network from the plot of storage (*G*′) and loss modulus (*G*″) against temperature showed 84, 79, and 49 min for EP, EP-PEAG, and EP-DAG (Figure 5ii,iii,iv), respectively. The lower gel point of the monofunctional amine (DA, PEA)-incorporated system could be a result of the dual accelerating effect of the hydroxyl group supported by tertiary amines, whereas in EP-PEAG, only the tertiary amine is present for cure acceleration. The faster viscosity build-up of the EP-G system with the incorporation of PEA and DA is illustrated by the digital images showing the flow characteristics (Figure 5v) after the same interval of time.

### Thermomechanical Characteristics

The dynamic mechanical analysis (DMA) plot indicated a single *T*_g_ (131°C) for EP-G, whereas EP-DAG and EP-PEAG showed doublet peaks corresponding to two *T*_g_ values at 121 and 133 °C and 112 and 129 °C, respectively (Figure 5vi). The second *T*_g_ value of EP-DAG and EP-PEAG is closer to the *T*_g_ of EP-G, and thus, it can be attributed to the crosslinked network structures formed by the reaction of difunctional epoxy and difunctional amine groups. The first *T*_g_ noted in these cases indicated the formation of ether-bridged homopolymerized polymers through the reaction of monofunctional amines.

### Adhesive Characteristics

**Figure 6:**
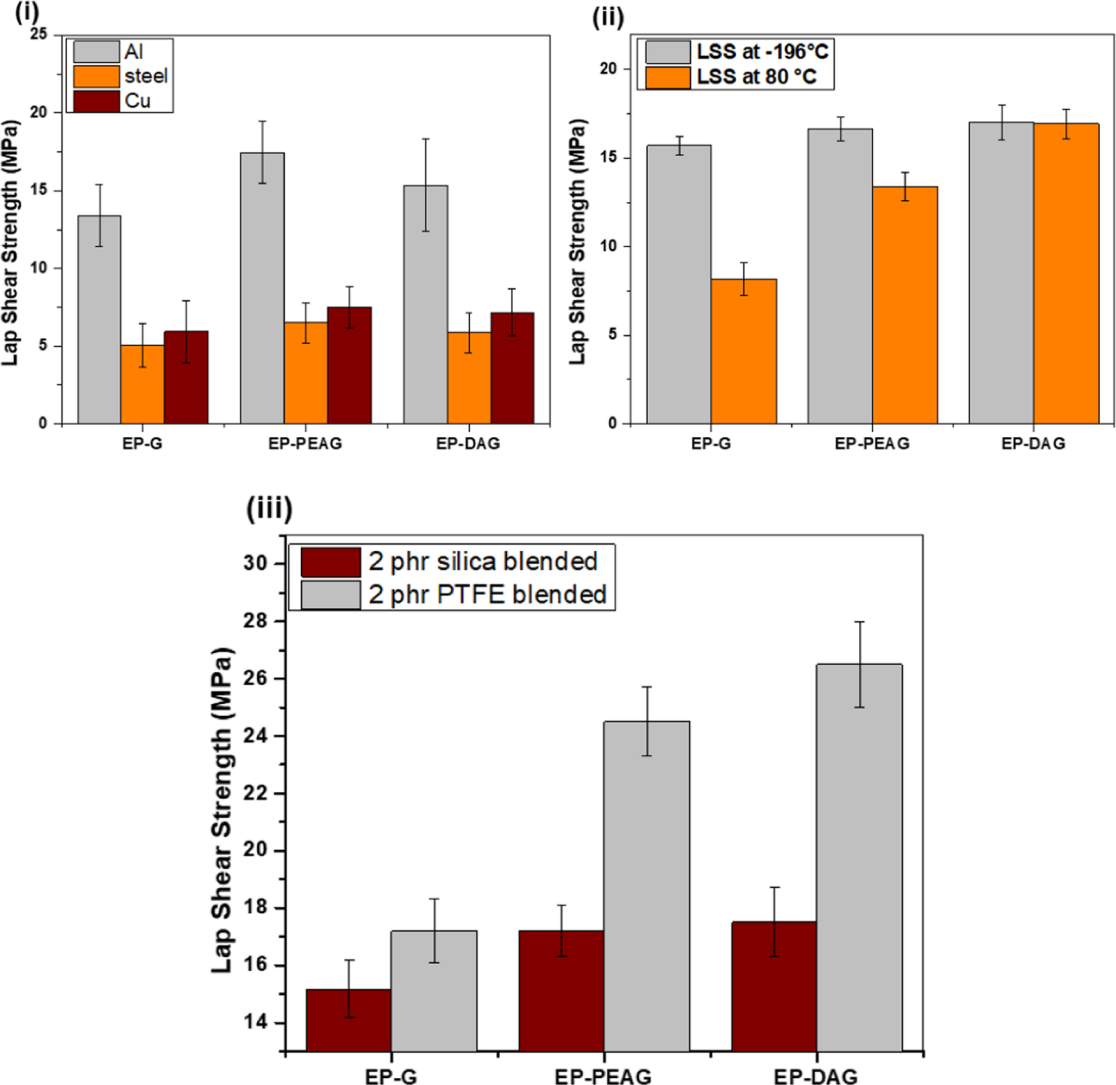
(i). LSS performance on different substrates. (ii) LSS evaluation at −196 and 80 °C. (iii) LSS performance of 2 phr silica- and PTFE-filled systems.

Adhesive properties based on lap shear strength (LSS) values were determined on different substrates, including Al, steel, and copper, using different formulations. The values are 13.7, 17, and 15 MPa on Al substrates for EP-G, EP-PEAG, and EP-DAG systems, respectively (Figure 6i). To determine the influence of etching as well as the roughening of Al coupons, LSS evaluation was carried out on coupons without chromic acid and without abrading. The results showed a reduction in LSS in the range of 47–51% for the coupons without etching, indicating the role of chromic acid etching and a fresh Al_2_O_3_ layer formation during etching. The coupon without abrading also showed a reduction in LSS values in the range of 65–68% for EP-G, EP-PEAG, and EP-DAG systems compared with Cr acid-etched specimens (Figure S9). EP-PEAG and EP-DAG possessed a higher adhesive strength than EP-G in all of the substrates on account of the formation of stiffer network structures as indicated by the enhanced modulus of the cured material (Figure S10).^41^ LSS values are correlated to the fracture toughness values for EP-PEAG, EP-DAG, and EP-G, which are found to be 2.33, 1.8, and 1.7 MPa M^1/2^, respectively. The crosslinking density, also calculated from the dynamic mechanic analysis, revealed a higher extent of crosslinking for EP-G and EP-DAG (1700 and 1300 moles/m^3^, respectively) than for EP-PEAG (890 moles/m^3^) (Figure S11). LSS evaluation at an elevated temperature (80°C) and under LN_2_ conditions (−196 °C) was also carried out on Al substrates (Figure 6ii). All of the systems maintained the adhesive strength at −196 °C, indicating the sustainability of the adhesives at cryogenic temperatures, which is a direct indication of their integrity with minimal variation in the tensile strain of the different systems (Figure S10). At 80 °C, EP-DAG exhibited better performance compared with EP-PEAG and EP-G systems on account of its comparatively higher *T*_g_ in addition to enhanced stiffness.

The incorporation of fillers such as silica nanoparticles and PTFE powders resulted in improved adhesive strength compared with the neat adhesive system because of the reinforcement effect of nano- and microfiller particles (Figure 6iii). The higher strength noted for the PTFE-incorporated system than the silica nanoparticle-incorporated system can be attributed to the improved toughness imparted by thermoplastic PTFE particles, and the results are corroborated by the mechanical property (tensile) evaluation (Table 3).^42,43^ The increased strength of EP-DAG and EP-PEAG systems could be due to the additional reinforcement effect offered by fillers in addition to the presence of stiffer crosslinking structures.

**Table 3:** Tensile Properties of PTFE- and Silica-Incorporated Adhesives

system	EP-G neat	EP-G-2 phr silica	EP-G-2 phr PTFE
tensile strength (MPa)	83 ± 0.2	42 ± 8	60 ± 10
modulus (GPa)	2.7 ± 0.1	2.6 ± 0.1	2.8 ± 0.1
percentage elongation	3.75 ± 0.05	3.1 ± 0.3	3.7 ± 0.6

### Moisture Resistance Studies

**Figure 7:**
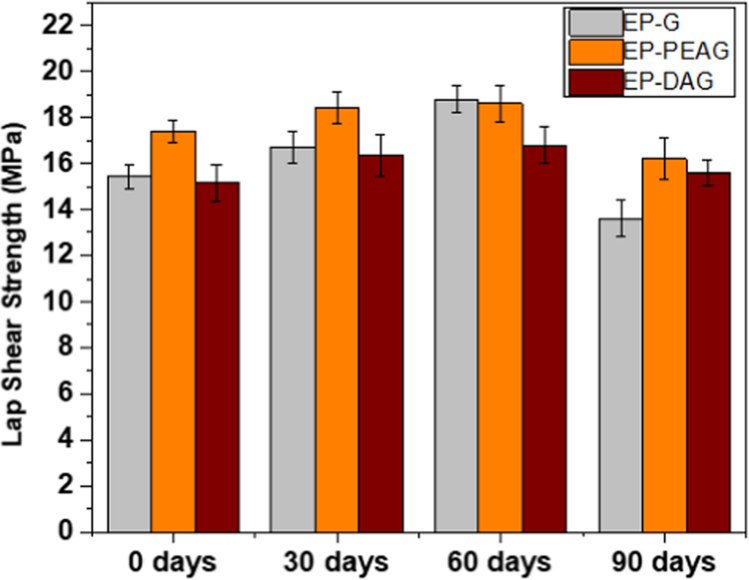
Lap shear strength of different systems after exposure to water for different durations.

The influence of moisture on bonded coupons was evaluated by exposing the bonded and cured specimens to distilled water for up to 90 days. Interestingly, after 30 and 60 days, a slight enhancement is noted in LSS, and then after 90 days, the system exhibited a marginal reduction in LSS values (Figure 7).

**Figure 8:**
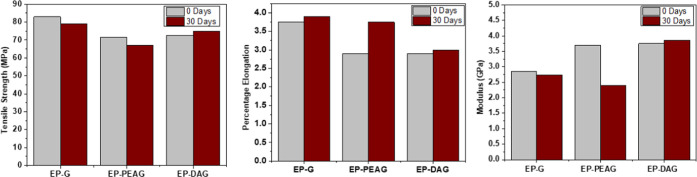
Tensile properties of EP-G, EP-PEAG, and EP-DAG after exposure to water.

Tensile property evaluation before (EP-G-0 and EP-PEAG-0) and after (30 days) exposure to moisture (EP-G-30 and EP-PEAG-30) resulted in a reduction of tensile strength and modulus with slight enhancement in the percentage elongation. This could be due to the moisture-induced plasticization effect on the cured adhesive network structure (Figure 8).^44,45^

Further, the gel point and swelling index (eqs 1 and 2 given in the Experimental Section) of the cured adhesives were determined by dipping in distilled water for different durations. The swelling index is a measure of water interaction with the cured network structures. After a period of 3 days, the swelling index of EP-G was lower compared with EP-PEAG and EP-DAG, indicating lower porosity of the cured networks of EP-G than those of EP-PEAG and EP-DAG (Figure S12), making it less vulnerable to plasticization through interaction with water. The swelling index and gel contents were also evaluated in chloroform as well as in glycerol by dipping the cured material in the respective solvents for 3 days. It was noted that the swelling index in chloroform solvent is higher because of the higher penetration ability of the chloroform solvent than glycerol and water (Table S3).

After 30 days of exposure to water, the swelling index of EP-G also increased, and for all of the systems, the total percentage of water absorption remained the same (Table 4). The gel content of the cured adhesive in water was also evaluated after 3 and 30 days of water exposure. After 3 days, the gel contents of EP-G, EP-PEAG, and EP-DAG were 99.4, 99.12, and 98%, and after 30 days, the values were 97.7, 96.6, and 98%, respectively, indicating a lower extent of interaction of water molecules with EP-DAG compared with the other systems on a longer exposure period. This observation is supported by the mechanical property evaluation (refer Figure 8). The EP-DAG system showed comparable tensile properties before and after 30 days of exposure to water. The improved performance of EP-DAG can be attributed to the influence of catechol amine moieties facilitating cohesive interactions by reaction with epoxy groups.^46^

**Table 4:** Gel Content and Swelling Index of Samples

	EP-G		EP-PEAG		EP-DAG	
properties	3 days	30 days	3 days	30 days	3 days	30 days
gel content (%)	99.4	97.7	99.12	96.6	98	98
swelling index (%)	0.4	2.26	1.7	0.6	1.98	0.49

### Underwater Bonding Performance of the Adhesive Formulations

**Figure 9:**
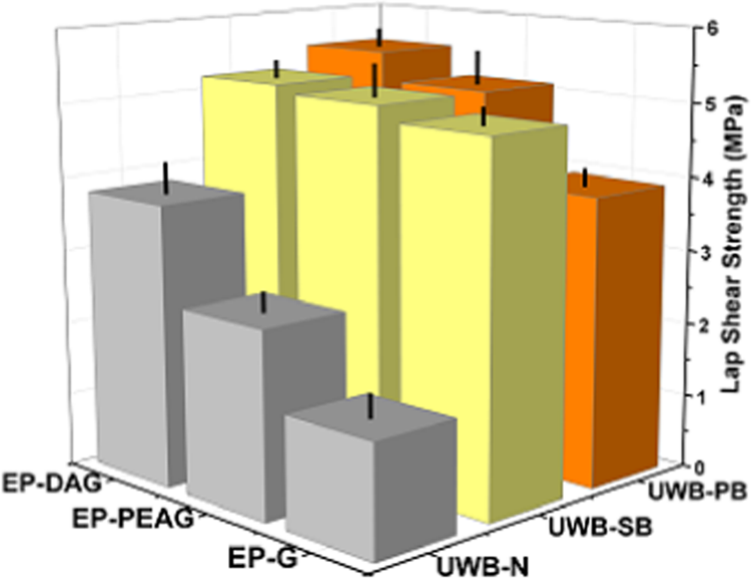
LSS of the neat adhesive, nanosilica-blended, and PTFE-blended samples under distilled water conditions.

Underwater bonding trials were carried out on an Al substrate for the adhesive as such (neat resin, represented as UWB-N in Figure 9) under distilled water conditions, resulting in an LSS reduction of about 90% for EP-G systems, 86% for EP-PEAG, and 75% for EP-DA compared with their adhesive performance under ambient conditions. Interestingly, the percentage of reduction is more prominent in the neat system compared with the modified system, emphasizing the impact of modification of epoxy. Further to this, EP, EP-DA, and EP-PEA systems were blended with (2 phr) nanosilica and PTFE powders (represented as UWB-SB and UWB-PB, respectively, in Figure 9), and LSS was determined. LSS results were similar for all of the formulations (4.1–5.5 MPa). Although the LSS under dry conditions was found to be higher for EP-PEAG, the underwater LSS values were similar for all of the systems. The results indicated that the blended system exhibited similar LSS values irrespective of the system chosen, with the retention of LSS values (30–33%) for all of the formulations. This is an important observation to prove the role of silica and PTFE fillers in maintaining the adhesive performance under underwater conditions by partially preventing the mixing of fillers of the adhesive with water because of the increased resin mix viscosity.

### Formation and Characterization of Liquid Marbles

**Figure 10:**
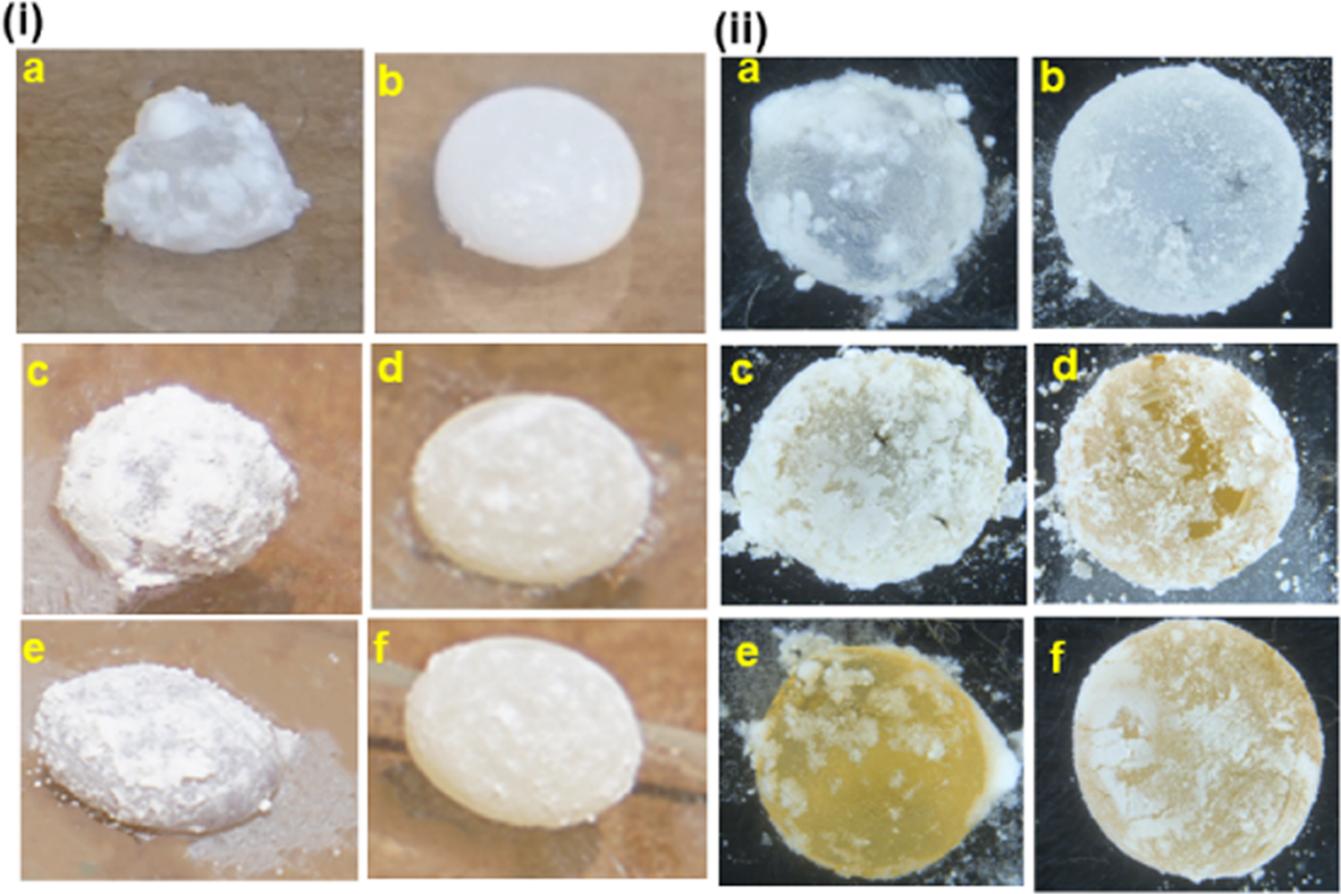
(i). Digital images of EP-G, EP-PEAG, and EP-DAG silica liquid marbles (a, c, and e) and PTFE liquid marbles (b, d, f), respectively. (ii) Optical images of EP-G, EP-PEAG, and EP-DAG silica liquid marbles (a, c, e) and PTFE liquid marbles (b, d, f), respectively.

An innovative concept, i.e., liquid marble (LM) technology, was proposed for effecting underwater bonding. Liquid marbles were prepared by dropping an adhesive mix (3 mL) in a bed of silica and PTFE powder separately; upon gentle shaking, a thin layer of powder was wrapped on the adhesive droplet, forming a hydrophobic particle boundary. Adhesive marbles formed using nanosilica exhibited nonuniform and incomplete overwrapping of powders, whereas the PTFE powder formed a uniform outer layer. The PTFE-powder-wrapped adhesive drops exhibited an almost spherical shape irrespective of the functionalization of the adhesive because of the higher hydrophobicity of PTFE than that of silica-wrapped marbles, which formed quasi-spherical marbles with a distorted surface finish (Figure 10). The amount of silica particles required for the formation of liquid marbles based on EP-G, EP-PEAG, and EP-DAG is calculated by composite and gravimetric methods (SI page No. 9–13 and Table S4). The coating thickness is estimated based on the difference in the volume of the resin droplet and the corresponding marble volume. The coating layer thicknesses (μm) are estimated to be 6.87, 9, and 10.32 μm for EP, EP-PEA, and EP-DA silica marbles, respectively. The PTFE-based liquid marble showed slightly lower coating thicknesses of 3.8, 4.6, and 9.39, respectively, for EP, EP-PEA, and EP-DA PTFE LMs. The higher coating thickness of silica nanoparticles is attributed to their smaller particle size and hydrophilic characteristics compared with PTFE, which enable better resin–filler interaction, leading to a higher particle population on the boundary.^47^ The formation of a nonuniform coating with agglomerated silica particles noted for silica-LM (Figure 10) is also a factor responsible for the higher coating thickness. The formation energy (Δ*G*) of liquid marbles is calculated using eq 5
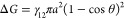
5where γ_12_ is the surface energy of the liquid and *a* is the radius of the particle. The PTFE- and silica-based LMs showed Δ*G* values of 11.02–11.07 × 10^–15^ and 2.9–3.6 × 10^–17^ J, respectively. The higher formation energy of PTFE-based liquid marbles is attributed to its more hydrophobic nature and higher particle size compared with silica particles.^48^

**Figure 11:**
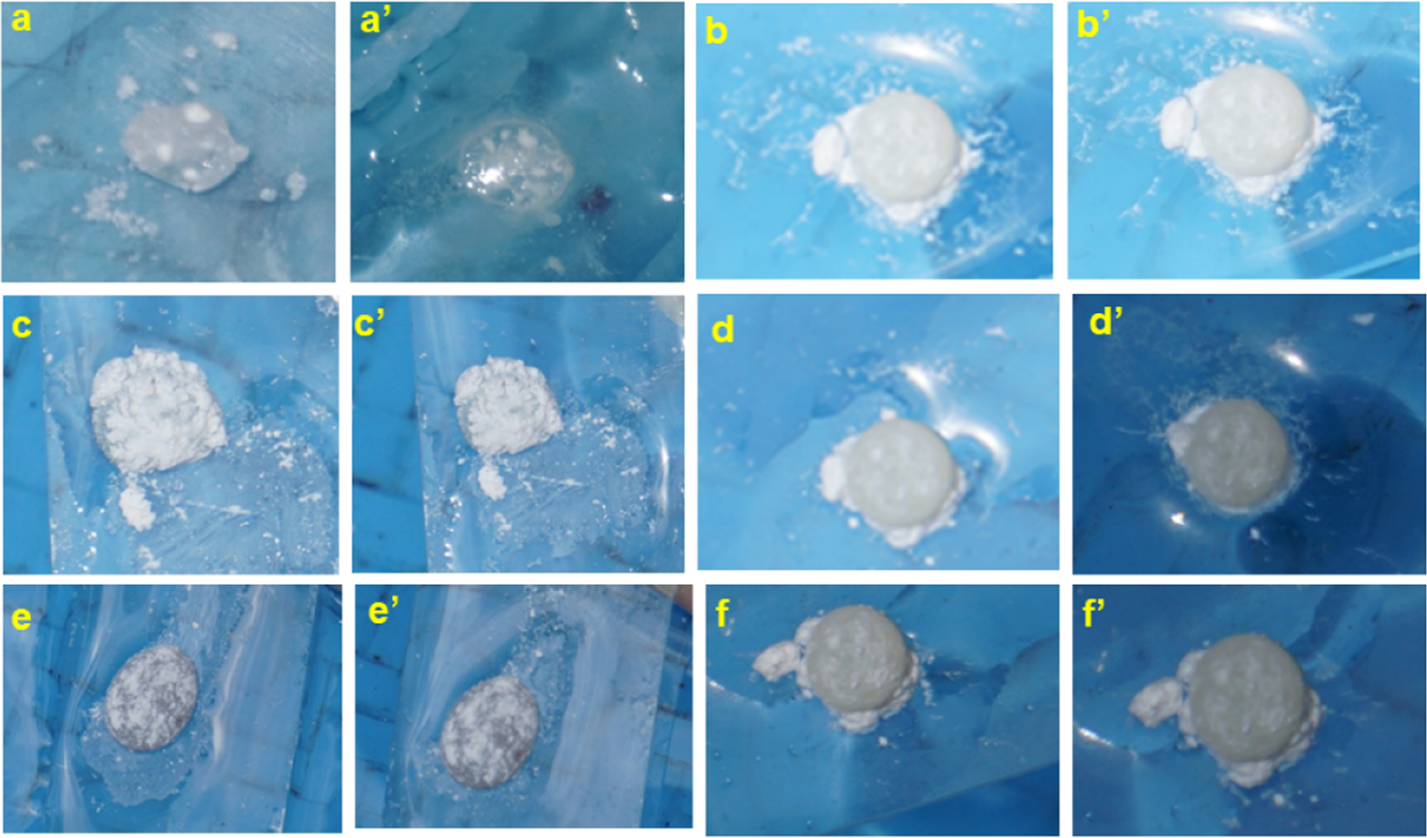
Digital images of EP-G, EP-PEAG, and EP-DAG silica liquid marbles exposed to water for 1 min (a, c, e) and 2 min (a′, c′, e′), respectively. PTFE LM after exposure to water for 1 min (b, d, f) and 2 min (b′, d′, f′), respectively, for EP-G, EP-PEAG, and EP-DAG.

The stability of the adhesive marbles was evaluated by dipping them in water for 1 and 2 min, which proved to be more detrimental for silica marbles than for PTFE marbles (Figure 11). The PTFE-wrapped particles maintained their integrity and shape after dipping in water, indicating the role of the more hydrophobic barrier. Mechanical property evaluation of the bulk liquid marble is not possible as the marble is highly fragile in nature. However, using a rheometer, the elastic force and maximum deformation before breakage were calculated using eq 6
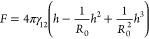
6where *F* is the elastic force, *h* is the deformation, *R*_0_ is the actual radius of the marbles, and γ_12_ is the effective surface tension of the liquid inside the marbles. In the present case, among the three systems studied, EP-G showed a lower deformability and elastic force because of its lower viscosity characteristics compared with EP-PEAG and EP-DAG (Table S5).

The energy required for breaking the marble is also important. As the energy for breaking is expected to be very low, the determination process is facilitated by the potential energy analogy. The marbles were dropped manually from different heights on an aluminum surface, and the maximum dropping height required for the marble to burst was noted. For measurement accuracy, the volume of liquid inside the marble is kept constant at 5 μL. The potential energy of marbles at a particular height is given by the equation *E* = *mgh* (where *m* is the mass of the marble, *g* is the gravitational acceleration, and h is the dropping height). PTFE-based liquid marbles showed a higher potential energy (Table S6), revealing higher stability compared with silica-based liquid marbles.

### Underwater Bonding Using Liquid Marbles

**Figure 12:**
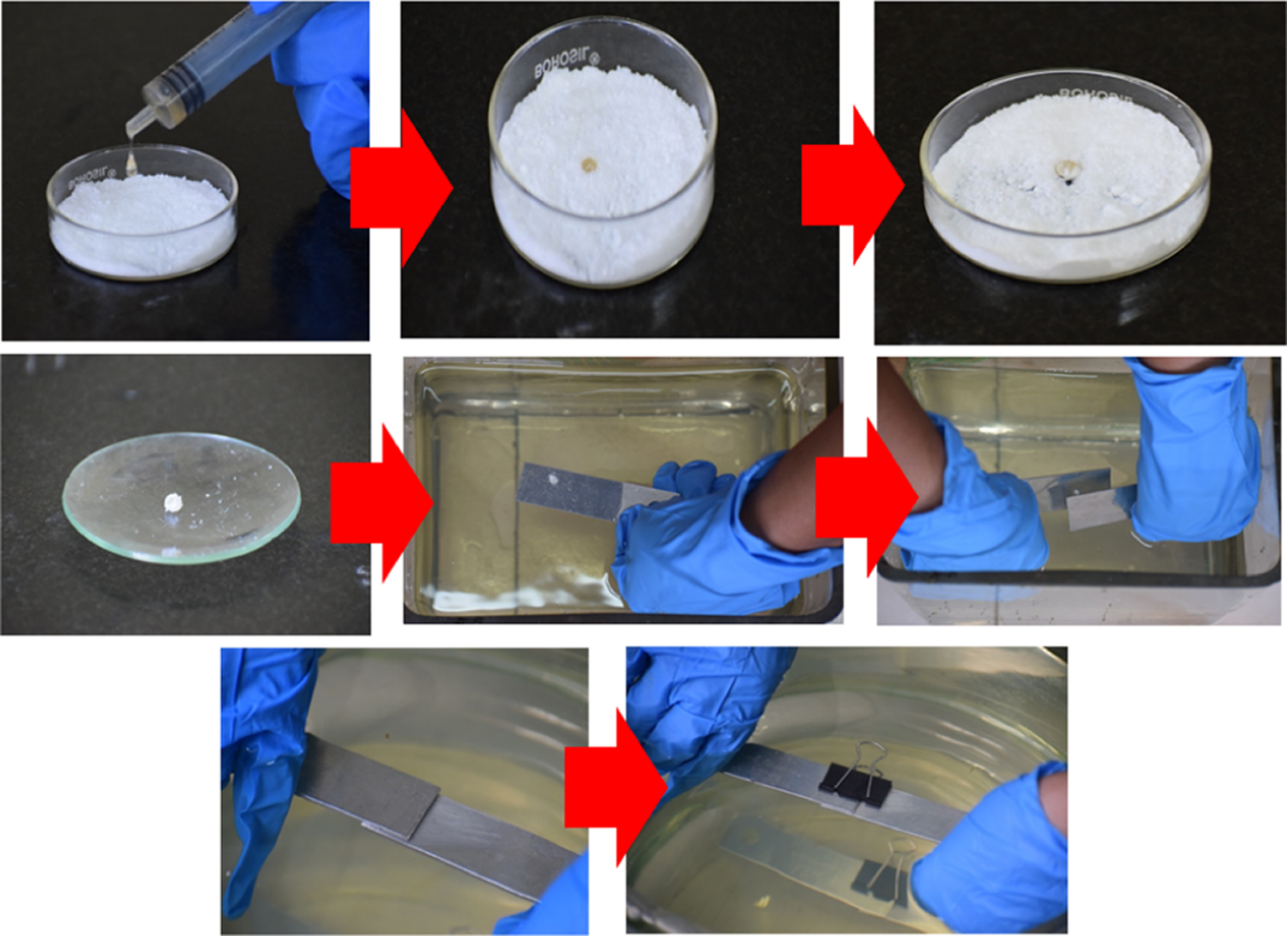
Sequence of adhesive marble formation and its underwater bonding.

The sequence of marble formation and its application for underwater bonding are shown in Figure 12.

**Figure 13:**
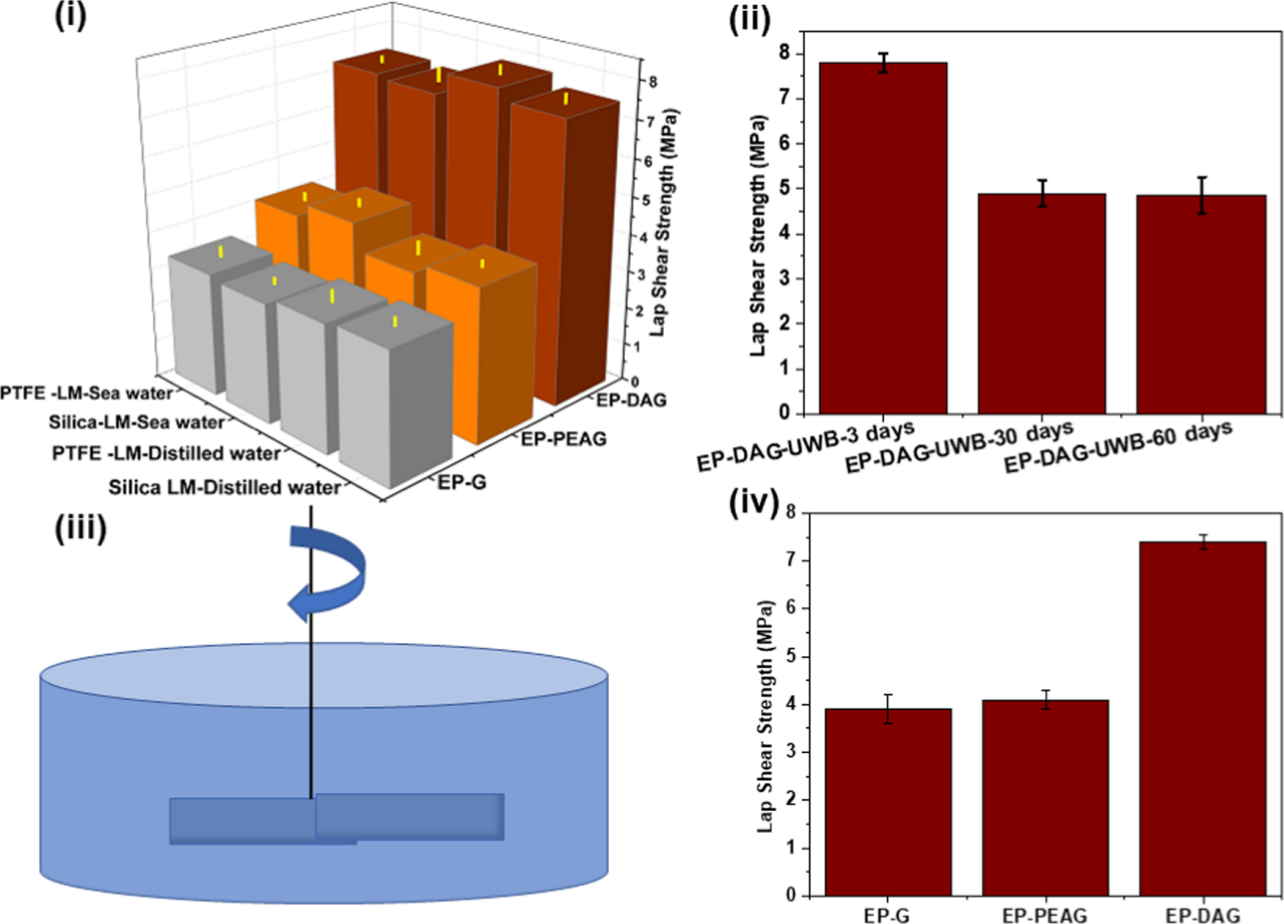
(i) LSS of samples bonded using liquid marble technology (LMT) after exposing the coupons to distilled water for 3 days. (ii) LSS after exposure of samples bonded using LMT underwater for 60 days. (iii) Schematics of the stirring experiment. (iv) Results of LSS evaluation after the stirring experiment.

Underwater bonding (UWB) was carried out using adhesive marbles under distilled water and seawater, followed by curing for 3 days underwater. The adhesive performance (Figure 13i) of EP-DA was found to be higher (7.5 MPa) compared with those of EP-G and EP-PEAG systems, which exhibited 3.2–4.5 MPa strength, respectively, after curing. This may be attributed to multiple factors that influence the adhesive performance, such as higher viscosity, fast curing, and higher strength than other adhesive marbles of EP-PEAG and EP-G. Liquid-marble-based UWB showed higher adhesive strength than the filled system, emphasizing that the PTFE- and silica-filler barrier help prevent the mixing of the adhesive with water. A detailed literature review in the relevant domain is carried out, and the adhesive strength of the current system vis-à-vis other systems is presented in Table S7. The value reported in the present study is the highest for an underwater bonding adhesive, comparable to the primer-based adhesives reported in our previous report.^34^ No appreciable change is noted for the specimens bonded under seawater and distilled water. The durability of the adhesive joint after UWB was evaluated by keeping the bonded coupons in distilled for different durations up to 60 days, and the specimens were tested at intervals of 30 days (Figure 13ii). The performance was compared on steel and Cu specimens also under underwater conditions, and the results showed a decrease in the LSS percentage compared with that under dry conditions (Figure S13). In addition, the performance of the adhesive joint under harsh marine conditions is also demonstrated at the laboratory level. For this, the cured specimens were stirred using a mechanical motor at an rpm of 150 for 12 h under underwater conditions (Figure 13iii). It is noteworthy that the adhesive joint for all of the systems was intact, with no reduction in LSS values (Figure 13iv) after the stirring experiment.

The failure mode of the debonded specimens indicated a mixed adhesive and cohesive failure when tested after 3 days of curing, whereas the specimens dipped in water for 60 days showed adhesive failure. This indicated the corrosion of the Al surface on prolonged water exposure, leading to the weakening of the Al–adhesive interface.

## Conclusions

In this study, epoxy adhesives incorporated with monofunctional amines with and without catechol were realized. The role of catechol in accelerating cure kinetics, thus enabling a higher viscosity build-up and adhesive strength, was demonstrated. The DA- and PEA-incorporated systems showed a higher activation energy compared with the EP-G system. The decrease in the gel point duration and faster viscosity build-up for EP-DAG and EP-PEAG systems also confirmed the curing acceleration effect by DA and PEA. The adhesive performance was evaluated in three ways, viz., neat resin, blended with fillers, and liquid marble method. The adhesive strength, determined under ambient dry conditions on different substrates, showed similar adhesive performance for all of the systems. Among neat adhesives, the underwater bonding strength was higher for EP-DAG (3.8 MPa), whereas EP-PEA and EP-G systems exhibited 2.2 and 1.5 MPa, respectively. On blending with fillers, all systems showed a similar adhesive strength of 4.1–5.5 MPa. In the liquid marble form, EP-DAG showed an excellent adhesive strength of 7.3–7.8 MPa, whereas EP-G and EP-PEAG exhibited values of 3.2–3.5 and 3.8–4.5 MPa, respectively, emphasizing the critical role of a coacervate formation for imparting higher adhesive strength under wet conditions.

## Supporting Information Available

Scheme S1: Schematic representation of EP-PEA and EP-DA systems. Figure S1: Zoomed portion of FTIR spectra of EP-DAG, EP-PEAG, and EP-G for peaks at (a) 1509 and (b) 915 cm^–1^; Figure S2: FTIR spectra of EP-G, EP-PEAG, and EP-DAG after storage of 90 days; Figure S3: ^1^ H NMR spectra of (a) EP, (b) DA, (c) EP-DA, (d) PEA, and (e) EP-PEA; Figure S4: DSC thermograms of EP-G, EP-PEAG, and EP-DAG at different heating rates; Figure S5: (i) DSC and (ii) FTIR curves of cured samples of EP-G, EP-PEAG, and EP-DAG; Figure S6: FTIR of the EP-G system at 60 °C recorded at different time periods of (a) 0, (b) 20, and (c) 45 min; Figure S7: optimized structure of the EP-G adhesive; Figure S8: TGA thermograms of different systems; Figure S9: LSS of adhesives on Al coupons; Figure S10: tensile properties such as (i) percentage elongation, (ii) tensile strength, and (iii) modulus of EP-G, EP-PEAG, and EP-DAG; Figure S11: storage modulus of EP-G, EP-PEAG, and EP-DAG systems; Figure S12: optical Images of (i) EP-G, (ii) EP-PEAG; and (iii) EP-DAG without abrading and Cr acid etching; Figure S13: LSS on steel and Cu using EP-G, EP-PEAG, and EP-DA. Table S1: molecular weight determination; Table S2: CHN percentages of EP, EP-PEA, and EP-DA; Table S3: swelling index and gel content of EP-G, EP-PEAG, and EP-DAG in solvents; Table S4: thickness of coating calculated by the composite method; Table S5: elastic force determination of samples; Table S6: energy of bursting of different systems; and Table S7: underwater adhesion strength comparison of different adhesives. Equation S1: crosslinking density calculation. Calculation S1: calculation of the thickness of silica- and PTFE-coated epoxy marbles (Page S9–S13) (PDF)
