# Supplementary material for: Catechol-Amine-Decorated Epoxy Resin as an Underwater Adhesive: A Coacervate Concept Using a Liquid Marble Strategy
Source: ACS Omega. 2023 Feb 16;8(8):7289–301. doi: 10.1021/acsomega.2c04163 (PMC9979230; doi:10.1021/acsomega.2c04163)
Supplement: Supplementary file 1 — ao2c04163_si_001.pdf [file ao2c04163_si_001.pdf]

Catechol Amine Decorated Epoxy Resin as Underwater Adhesive; A Coacervate Concept Using Liquid Marble Strategy

Monisha Baby, [†,£] *Soumyamol Panthaplackal Bhaskaran,* [‡] *and Satheesh Chandran Maniyeri**

£

[†]Cochin University of Science and Technology, Ernakulam 682022, Kerala, India.

[‡]Analytical and Spectroscopic Division and [£]Polymers and special Chemical Division,

Vikram Sarabhai Space Centre, Thiruvananthapuram 695022, Kerala, India.

Keywords: (mussel analogue, low surface energy, adhesion promoter, hybrid morphology

)

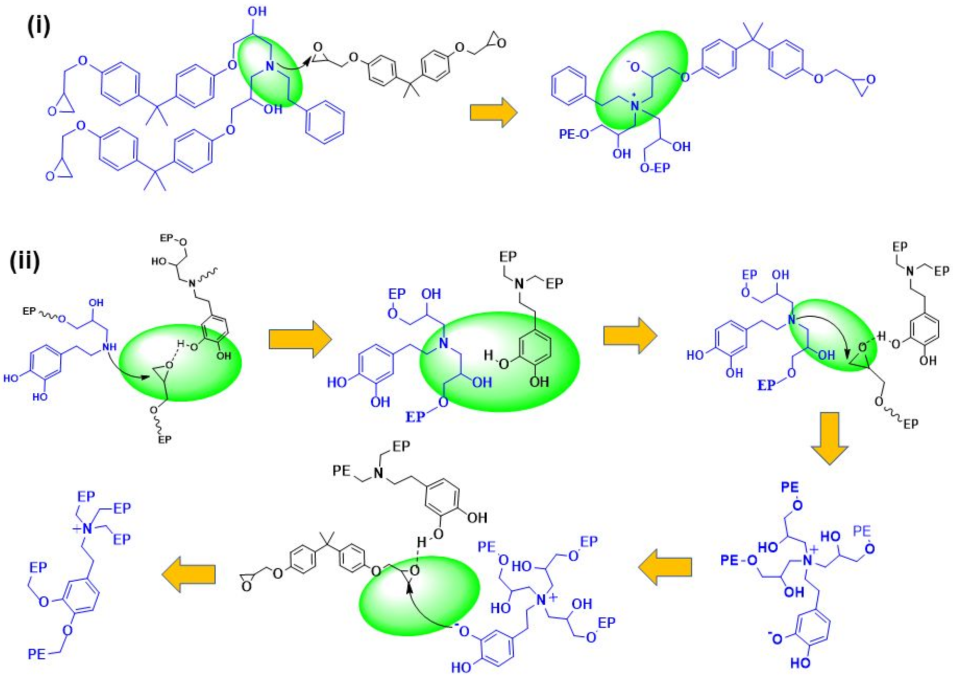

Scheme S1: Schematic representation of EP-PEA and EP-DA system.

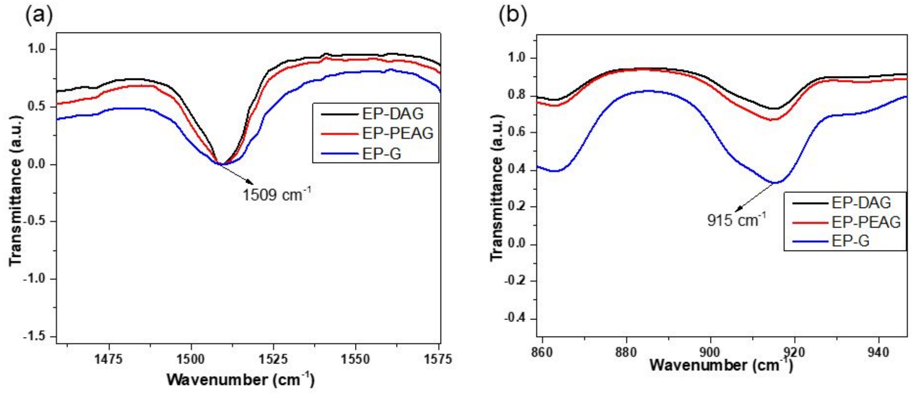

Figure S1: Zoomed portion of FTIR spectra of EP-DAG, EP-PEAG and EP-G for peaks at (a) 1509 and (b) 915 cm^{-1}.

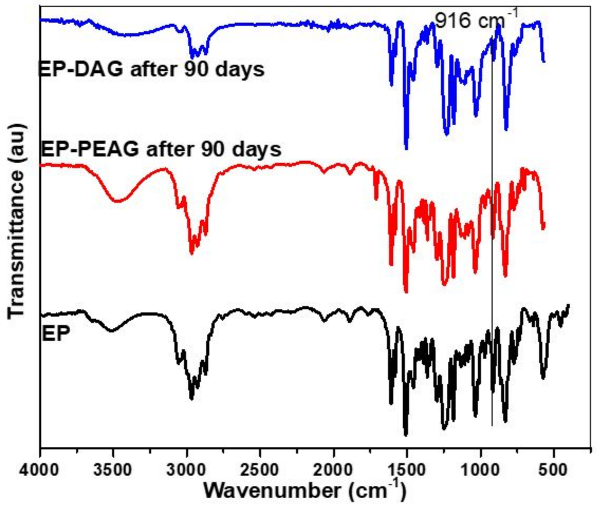

Figure S2: FTIR spectra of EP-G, EP-PEAG and EP-DAG after storage of 90 days.

Table S1: Molecular weight determination

System	Mn (g/mol)	PDI
EP	246	1.2
EP- PEA	896	1.5
EP-DA	1774	1.1

Table S1: CHN percentages of EP, EP-PEA and EP-DA

System	C (%)	H (%)	N (%)
EP	74.07	7.8	0
EP- PEA	73.4	7.0	1.4
EP-DA	72.3	7.1	1.3

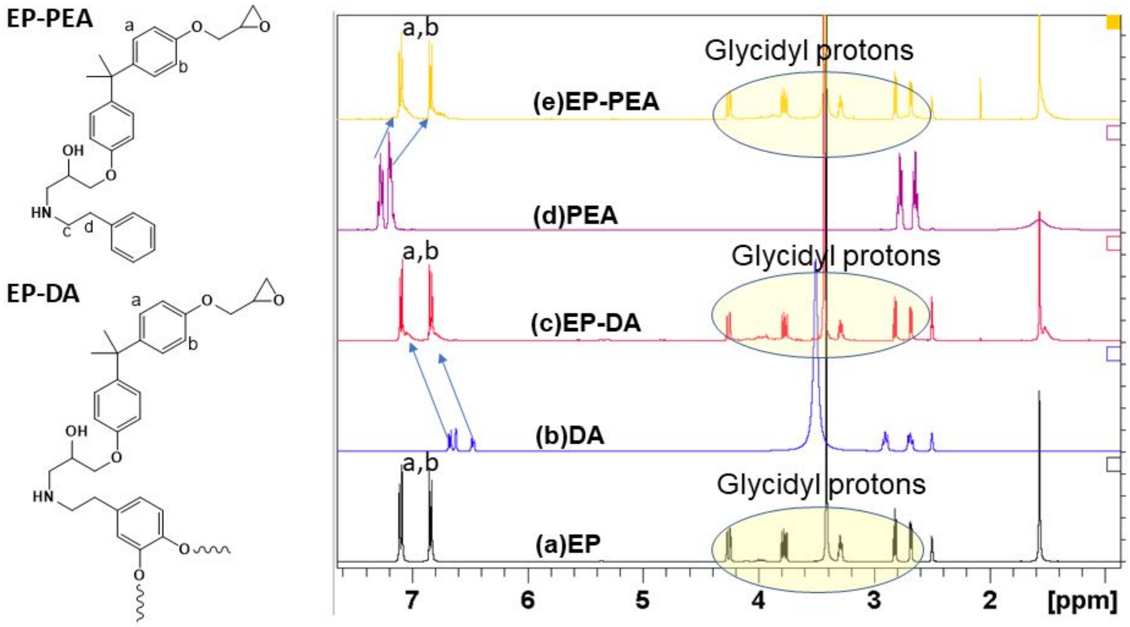

Figure S3: [1] H NMR spectrum of (a) EP, (b) DA, (c) EP-DA, (d) PEA and (e) EP-PEA.

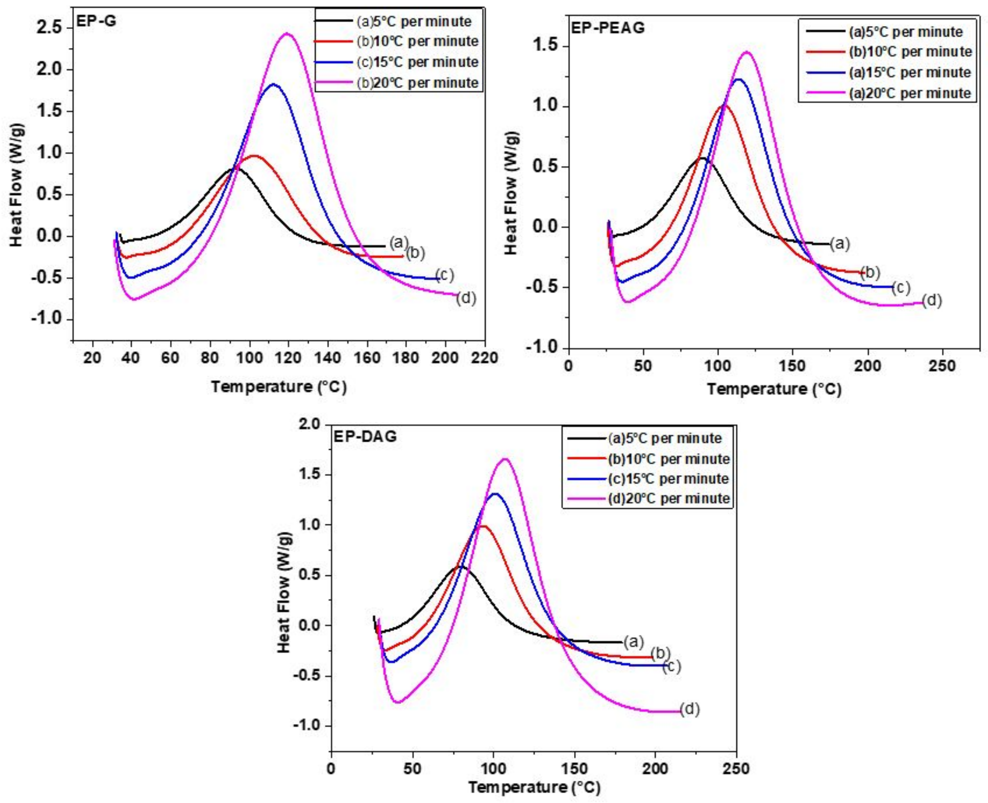

Figure S4: DSC thermogram of EP-G, EP-PEAG and EP-DAG at different heating rates.

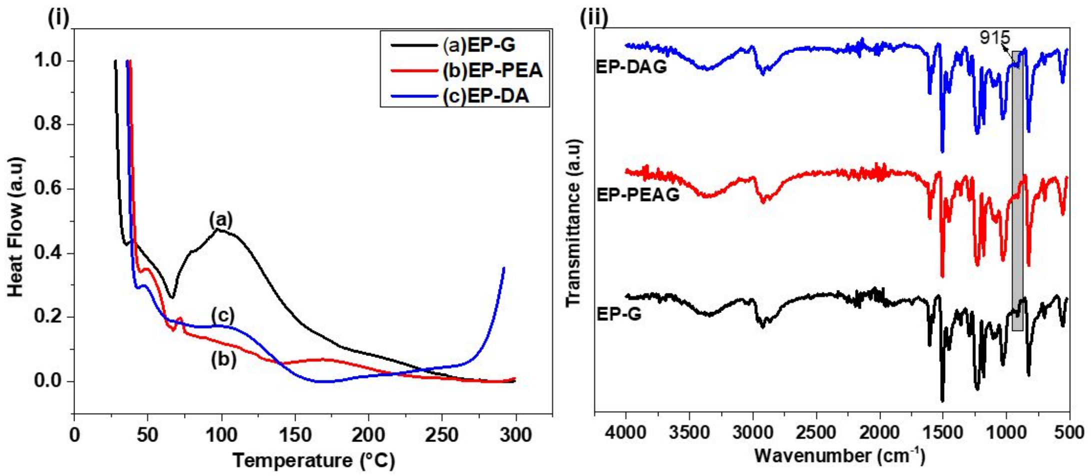

Figure S5: (i) DSC and (ii). FTIR curves of cured samples of EP-G, EP-PEAG and EP-DAG.

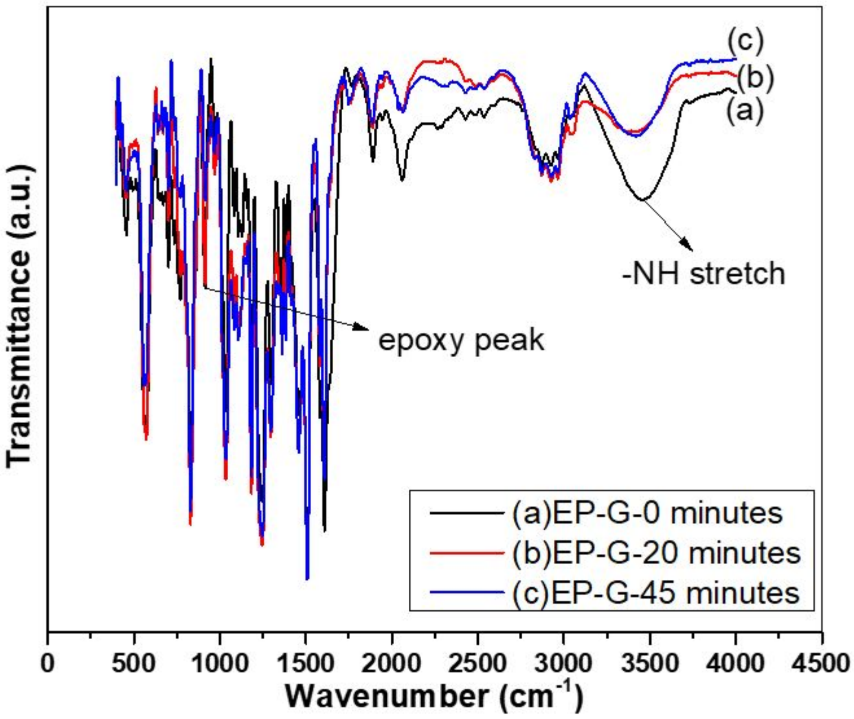

Figure S6: FTIR of EP-G system at 60 °C recorded at different time duration (a). zero, (b). 20 and (c). 45 minutes.

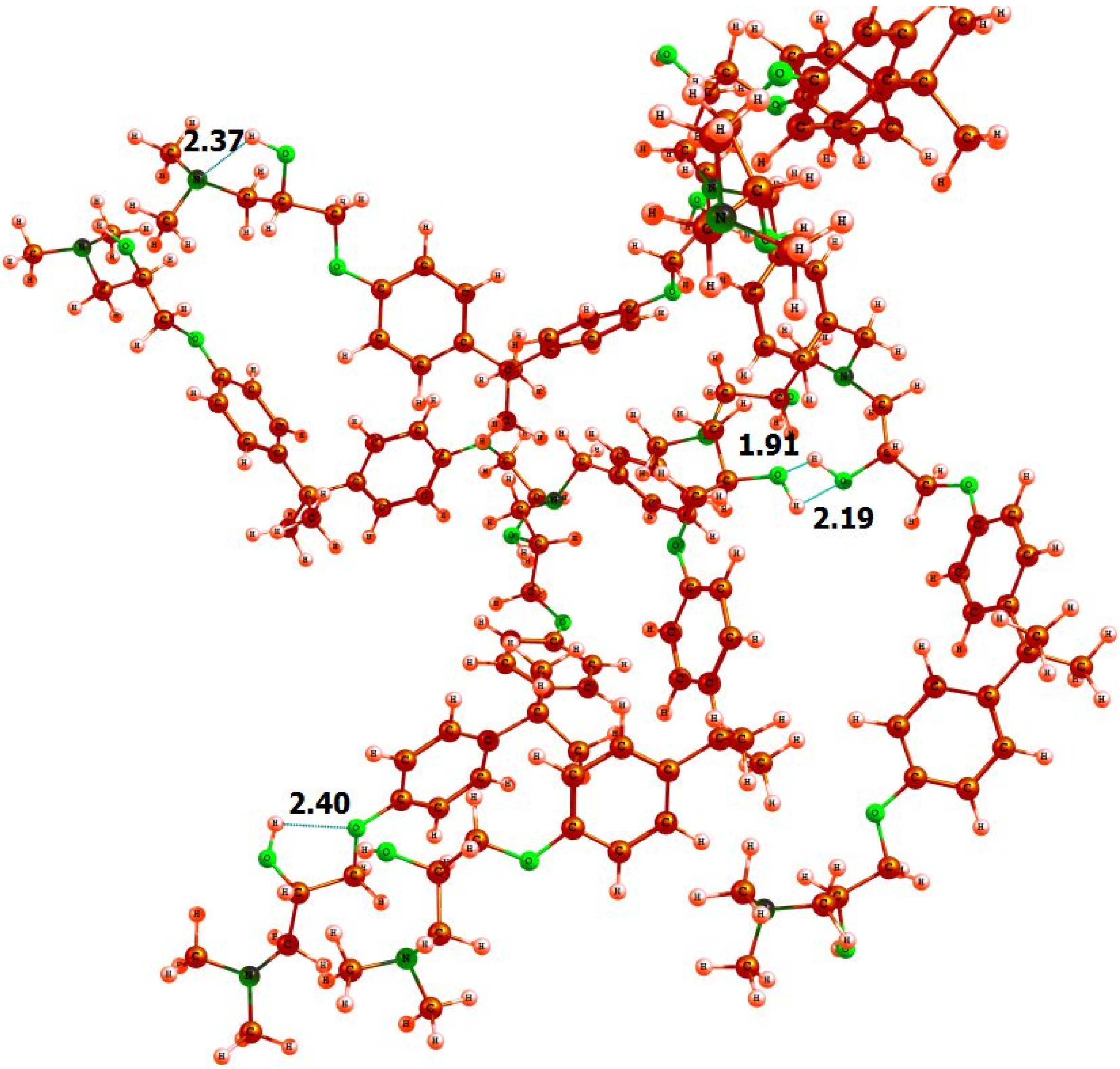

Figure S7: Optimized structure for EP-G adhesive.

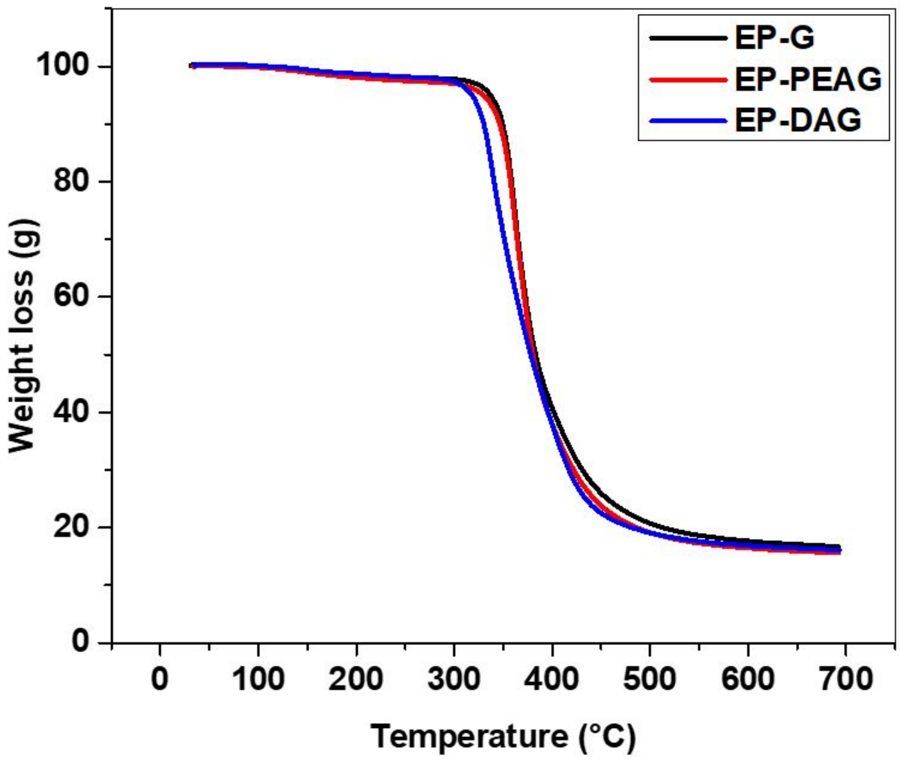

Figure S8: TGA thermogram of different systems.

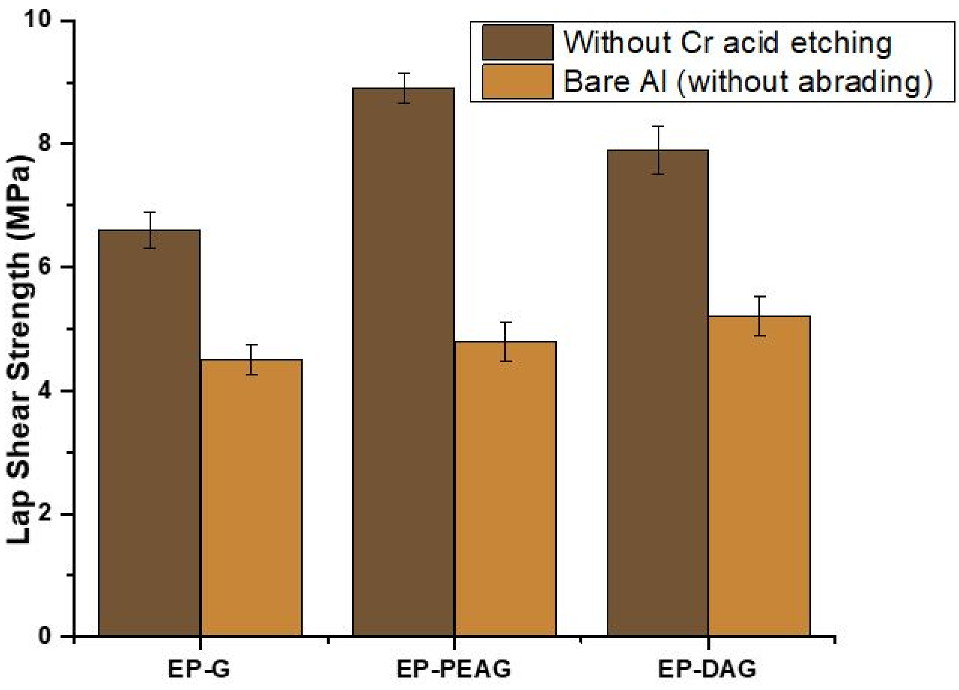

Figure S9: LSS of adhesives on Al coupons.

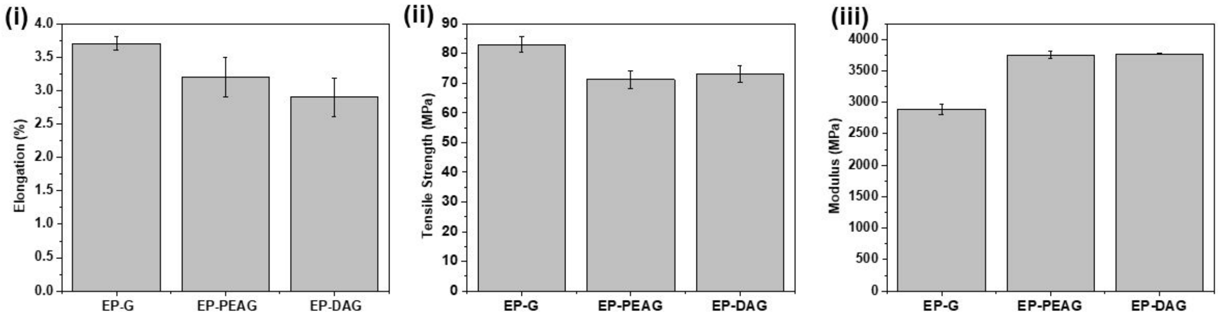

Figure S10: Tensile properties like (i). percentage elongation, (ii). tensile strength and (iii). modulus of EP-G, EP-PEAG and EP-DAG.

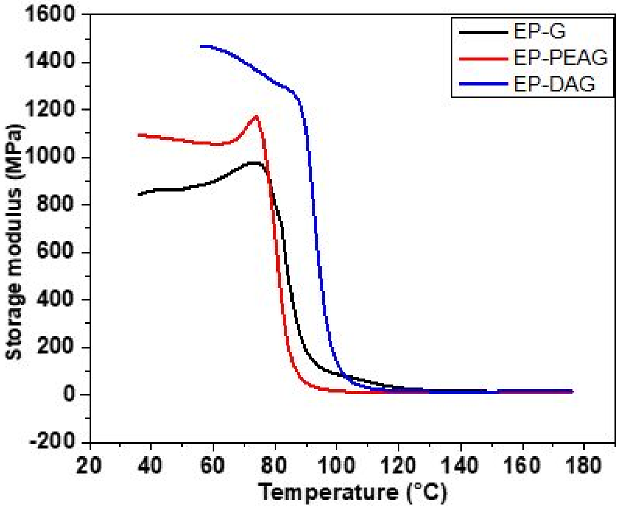

Figure S11: Storage modulus of EP-G, EP-PEAG and EP-DAG systems.

Crosslink density can be calculated from Equation S1 using DMA technique.

$\upsilon_e = E'/3RT$ ---------S1

Where,

υ_e –Crosslink density

E'-Storage Modulus

T-Temperature (T+20°C) in K corresponds to E'

Table S3: Swelling index and gel content of EP-G, EP-PEAG and EP-DAG in solvents

	Properties	EP-G	EP-PEAG	EP-DAG
		**3 days**	**3 days**	**3 days**
Chloroform	Gel content (%)	98.2	98.12	98
	Swelling Index (%)	5	15	4
Glycerol	Gel content (%)	99.85	99.94	99.92
	Swelling Index (%)	0.3	0.4	0.25

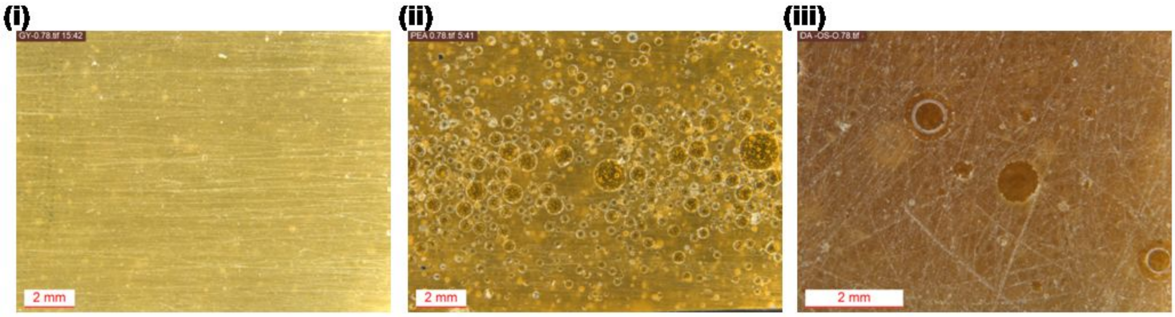

Figure S12: Optical Images of (i). EP-G, (ii). EP-PEAG and (iii). EP-DAG without abrading and Cr acid etching.

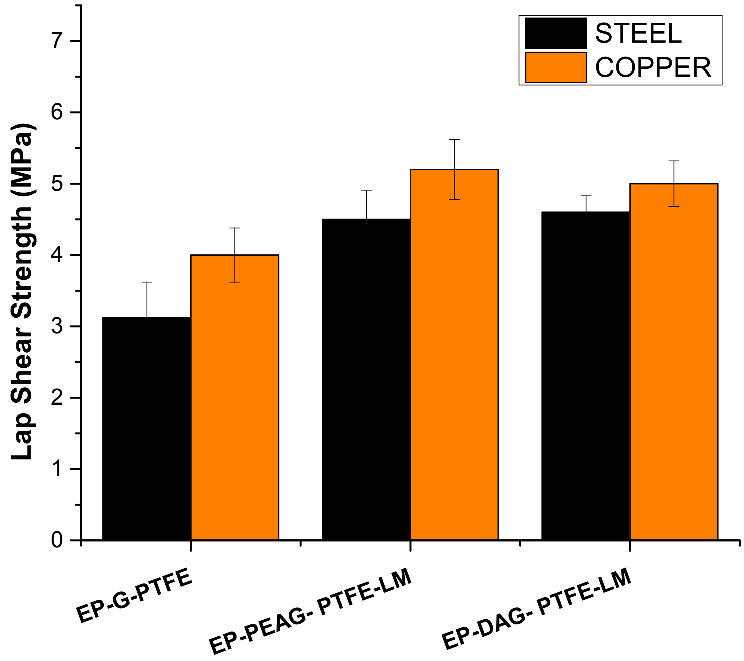

Figure S13: LSS on steel and Cu using EP-G, EP-PEAG and EP-DAG based PTFE LM.

Calculation S1: Calculation for the thickness of silica and PTFE coated epoxy marbles

i). Thickness of particles coating on EP by PTFE nanoparticles

a. By the composite method

Volume of EP droplet =10µL
Mass of EP droplet =0.0123 g
Density of liquid EP droplet (ρ_{EP}) =1.17 g/mL

Volume of EP droplets (V_{EP})=10.512µL
Volume of EP droplet (V_{EP}) =4/3 π r^3
Radius of EP droplet (r) =1.35916 mm;
Mass of 10µL EP marbles = 0.0125 g
Mass of coated PTFE= mass of marbles – mass of EP droplet = 0.0002g
Density of PTFE nanoparticle (ρ_{PTFE}) = 2.2g/cm^3
Volume of PTFE nanoparticles required to form 10µL EP = mass of PTFE/ density of PTFE=0.090µL
Volume fraction of PTFE to form marbles = 0.008919
Volume fraction of EP to form marbles= 0.9910
Equivalent density of marbles = $\rho_{\mathrm{EP}} \times V_{\mathrm{EP}} + \rho_{\mathrm{PTFE}} \times V_{\mathrm{PTFE}}$
= 1.17×0.9910+2.2×0.008919
= 1.179 g/mL
Volume of marbles = mass of marbles / equivalent density of marbles= 10.60136µL
Volume of marbles = 4/3 π R^3

Radius of marbles (R) = 1.3630 mm
Thickness of PTFE particle coating (t) = R – r =3.8μm

b. Gravimetric Method

Total Volume of EP marble = volume of EP + volume of particles = 10 + 0.090 = 10.090μL

Volume of marbles = 4/3×π R^3

Radius of marbles = 1.3630 mm

Thickness = Radius of Marble – Radius of droplet Thickness = 3.8μm

ii) Calculation for the thickness of PTFE coated EP-PEA marbles by the composite method

Volume of EP-PEA droplet =10μL
Mass of EP-PEA droplet =0.016 g
Density of liquid EP-PEA droplet (ρw) =1.18 g/mL

Volume of EP-PEA droplets (V_{EP})=13.55μL
Volume of EP-PEAdroplet ($V_{EP\text{-}PEA}$) =4/3 π r3
Radius of EP-PEA (r) =1.4791 mm
Mass of 10μL EP-PEA marbles = 0.0163 g
Mass of coated PTFE= mass of marbles – mass of EP-PEA droplet = 0.0003g
Density of PTFE particle (ρ_{PTFE}) = 2.2g/cm^3
Volume of PTFE nanoparticles required to form 10μL EP-PEA = mass of PTFE/ density of PTFE =0.136μL
Volume fraction of PTFE to form marbles = 0.0134
Volume fraction of EP-PEA to form marbles= 0.98658
Equivalent density of marbles = $\rho_{EP\text{-}PEA} \times V_{EP\text{-}PEA} + \rho_{PTFE} \times V_{PTFE}$
= 1.18×0.98522+2.2×0.0134
= 1.1920 g/mL
Volume of marbles = mass of marbles / equivalent density of marbles= 13.673234μL
Volume of marbles = 4/3 π R3
Radius of marbles (R) = 1.483659 mm
Thickness of PTFEnanoparticle coating (t) = R – r =4.559 μm

b. Gravimetric Method

Total Volume of EP-PEA marble = volume of EP-PEA droplet + volume of particles = 10 + 0.136 = 10.136μL

Volume of marbles = 4/3×π R^3

Radius of marbles = 1.483659 mm

Thickness = Radius of Marble – Radius of droplet Thickness = 4.559μm

iii) Thickness of PTFE particles coating on EP-DA

a.By the composite method

Volume of EP-DA droplet =10µL
Mass of EP-DA droplet =0.0133 g
Density of liquid EP-DA droplet ($\rho_{\text{EP-DA}}$) =1.26 g/ml

Volume of EP-DA droplet ($V_{\text{EP-DA}}$)=10.55µL
Volume of EP-DA droplet ($V_{\text{EP-DA}}$) =4/3πr³
Radius of EP-DA droplet (r) =1.361016 mm
Mass of 10µL EP-DA marbles = 0.01375 g
Mass of coated PTFE= mass of marbles – mass of EP-DA droplet = 0.00045g
The density of PTFE particle (ρPTFE) = 2.2 g/cm³
Volume of PTFE particles required to form 10µL EP-DA = mass of PTFE/ density of PTFE=0.2045µL
Volume fraction of PTFE to form marbles = 0.02045
Volume fraction of EP-DA to form marbles= 0.97996
Equivalent density of marbles = $\rho_{\text{EP-DA}} \times V_{\text{EP-DA}} + \rho_{\text{PTFE}} \times V_{\text{PTFE}}$
= 1.26×0.9799+2.2×0.02045
= 1.2796 g/mL
Volume of marbles = mass of marbles / equivalent density of marbles= 10.77450

Volume of marbles = 4/3 π R³
Radius of marbles (R) = 1.37044 mm
Thickness of PTFEparticle coating (t) = R – r =9.386µm

b. Gravimetric Method

Total Volume of EP-DA marble = volume of EP-PEA droplet + volume of particles = 10 + 0.225 = 10.2045µL

Volume of marbles = 4/3×π R³

Radius of marbles = 1.37044 mm

Thickness = Radius of Marble – Radius of droplet Thickness = 9.386µm

iv) Thickness of silica nanoparticles coating on EP by the composite method

Volume of EP droplet =10µL
Mass of EP droplet =0.0133 g
Density of liquid EP droplet (ρ_{EP}) =1.17 g/mL

Volume of EP droplet (V_{EP})=11.36 µL
Volume of EP droplet (V_{EP}) =4/3 π r3
Radius of EP droplet (r) =1.3947 mm
Mass of 10µL EP marbles = 0.01365 g
Mass of coated silica nanoparticle= mass of marbles – mass of EP droplet = 0.00035g

Density of silica nanoparticle (ρ_{SN}) = 2.0g/cm^3
Volume of silica nanoparticle required to form 10µL EP = mass of silica/ density of silica =0.175µL
Volume fraction of silica nanoparticles to form marbles = 0.0171
Volume fraction of EP to form marbles= 0.982318
Equivalent density of marbles = $\rho w \times Vw + \rho_{SN} \times V_{SN}$
= 1.17×0.98280+2×0.017469
= 1.18 g/mL
Volume of marbles = mass of marbles / equivalent density of marbles= 11.527

Volume of EP droplet (V_{EP})
Volume of marbles = 4/3 π R3
Radius of marbles (R) = 1.4015 mm
Thickness of silica nano particle coating (t) = R – r =6.87µm

b. Gravimetric Method

Total Volume of EP marble = volume of EP-PEA droplet + volume of particles = 10 + 0.15909= 10.15909µL

Volume of marbles = 4/3×π R^3

Radius of marbles = 1.4015 mm

Thickness = Radius of Marble – Radius of droplet Thickness =6.87µm

v) Thickness of PTFE nanoparticles coating on EP-PEA by the composite method

Volume of EP-PEAdroplet =10 µL
Mass of EP-PEA droplet =0.0136 g
Density of liquid EP-PEA droplet ($\rho_{EP\text{-}PEA}$) =1.18 g/mL

Volume of liquid droplet ($V_{EP\text{-}PEA}$)=11.52
Volume of liquid droplet ($V_{EP\text{-}PEA}$) =4/3 π r3
Radius of EP-PEA droplet (r) =1.40129 mm
Mass of 10µL EP-PEA marbles = .0141
Mass of coated silica particle= mass of marbles – mass of EP-PEA droplet = 0.0005g
Density of PTFE= 2g/cm^3
Volume of PTFE required to form 10µL EP-PEA = mass of silica/ density of silica =0.25 µL
Volume fraction of PTFE to form marbles = 0.02439
Volume fraction of EP-PEA to form marbles= 0.967741
Equivalent density of marbles = $\rho_{EP\text{-}PEA} \times V_{EP\text{-}PEA} + \rho_{SN} \times V_{SN}$
= 1.18×0.9756+2×0.02439
= 1.199 g/mL
Volume of marbles = mass of marbles / equivalent density of marbles= 11.7500µL
Volume of marbles = 4/3 π R3

Radius of marbles (R) = 1.41055 mm
Thickness of PTFE nano particle coating (t) = R – r =9µm

b. Gravimetric Method

Total Volume of EP-PEA marble = volume of EP-PEA droplet + volume of particles = 10 + .25= 10.25µL

Volume of marbles = 4/3×π R^3

Radius of marbles = 1.41015 mm

Thickness = Radius of Marble – Radius of droplet Thickness = 9µm

vi) Thickness of PTFE nanoparticles coating on EP-DA by the composite method

Volume of EP-DA droplet =10 µL
Mass of EP-DA droplet =0.0162 g
Density of liquid EP-DA droplet ($\rho_{\text{EP-DA}}$) =1.26 g/mL

Volume of EP-DA droplet=12.857µL
Volume of EP-DA droplet ($V_{\text{EP-DA}}$) =4/3 π r3
Radius of EP-DAdroplet (r) =1.453529 mm
Mass of 10 µL EP-DA marbles = 0.01685 g
Mass of coated silica= mass of marbles – mass of EP-DA droplet = 0.00065g
Density of silica= 2g/cm^3
Volume of silica required to form 10µL EP-DA = mass of silica/ density of silica =0.325µL
Volume fraction of silica to form marbles = 0.03147
Volume fraction of EP-DA to form marbles= 0.9685
Equivalent density of marbles = $\rho_{\text{EP-DA}} \times V_{\text{EP-DA}} + \rho_{\text{SN}} \times V_{\text{SN}}$
= 1.26×0.9685+2×0.03147
= 1.2832 g/mL
Volume of marbles = mass of marbles / equivalent density of marbles= 13.1330µL
Volume of marbles = 4/3 π R3
Radius of marbles (R) = 1.4638mm
Thickness of PTFE nano particle coating (t) = R – r =10.32µm

b. Gravimetric Method

Total Volume of EP-DA marble = volume of EP-DA droplet + volume of particles = 10 + .325= 10.325µL

Volume of marbles = 4/3×π R^3

Radius of marbles = 1.4638mm

Thickness = Radius of Marble – Radius of droplet Thickness = 10.32µm

Table S4: Thickness of coating calculated by the composite method

Systems	Thickness of coating µm	
	PTFE	Silica nanoparticle
EP	3.8	6.87
EP-PEA	4.6	9
EP-DA	9.35	10.32

Table S5: Elastic force determination of samples

Samples	Weight of sample (mg)	Deformation (µm)	Elastic Force (mN)
EP-G	10	2500	1.3
EP-PEAG	10	2400	1.26
EP-DAG	10	2200	1.16

Table S6: Energy of bursting of different systems

Marble	Mass of Marbles (g)	Maximum burst height (cm)	Energy of bursting (µJ)
EP-DA- PTFE	0.09	300 cm	264
EP-DA- Slica	0.085	200 cm	166
EP-PEA- PTFE	0.083	220 cm	178
EP_PEA-silica	0.080	180 cm	141
EP-G- PTFE	0.058	30 cm	17
EP-G- Silica	0.054	10 cm	5

Table S7: Underwater adhesion strength comparison of different adhesives

**Adhesive Type**	**Substrate**	**Adhesive Strength (MPa)**	**Condition**
Mussel-inspired adhesive	Ceramic	0.880	Wet[1]
Mussel-inspired adhesive	Ceramic	0.345	wet[1]
Hydrogel adhesive	Metal	3.012	wet[1]
Polydiol citrate-glycidyl methacrylate based adhesive	Glass, polyethylene, copper, rubber and silica	0.04-0.08	underwater[2]
PDMS	PET	2.8	underwater[3]
Poly catechol-Styrene	Al	3.5	underwater[4]
poly(dopamine methacrylamide-co-methoxethyl acrylate-co-adamantane-1-carboxylic acid 2-(2-methyl-acryloyloxy)-ethyl ester)	Fe	2.78	underwater[1]

Epoxy-Amine	Mussel mimicking polymer primed Al	7.8	underwater[5]
EP-DAG -PTFE-LM (present work)	Mussel mimicking epoxy polymer adhesive	7.8	underwater

References

1. Gao, L.; Ma, S.; Bao, L.; Zhao, X.; Xiang, Y.; Zhang, Z.; Ma, Y.; Ma, Z.; Liang, Y.-m.; Zhou, F., Molecular Engineering Super-Robust Dry/Wet Adhesive with Strong Interface Bonding and Excellent Mechanical Tolerance. *ACS Applied Materials & Interfaces* **2022,** *14* (10), 12684-12692.
2. Wang, Z.; Zhao, J.; Tang, W.; He, T.; Wang, S.; He, X.; Chen, Y.; Yang, D.; Peng, S., Robust Underwater Adhesives Based on Dynamic Hydrophilic and Hydrophobic Moieties to Diverse Surfaces. *ACS Applied Materials & Interfaces* **2021,** *13* (2), 3435-3444.
3. Yan, Y.; Huang, J.; Qiu, X.; Zhuang, D.; Liu, H.; Huang, C.; Wu, X.; Cui, X., A strong underwater adhesive that totally cured in water. *Chemical Engineering Journal* **2022,** *431*, 133460.
4. North, M. A.; Del Grosso, C. A.; Wilker, J. J., High Strength Underwater Bonding with Polymer Mimics of Mussel Adhesive Proteins. *ACS Applied Materials & Interfaces* **2017,** *9* (8), 7866-7872.
5. Baby, M.; Periya, V. K.; Sankaranarayanan, S. K.; Maniyeri, S. C., Bioinspired surface activators for wet/dry environments through greener epoxy-catechol amine chemistry. *Applied Surface Science* **2020,** *505*, 144414.
